# Zero-shot reconstruction of mutant spatial transcriptomes

**DOI:** 10.1016/j.patter.2026.101521

**Published:** 2026-03-31

**Authors:** Yasushi Okochi, Takaaki Matsui, Shunta Sakaguchi, Takefumi Kondo, Honda Naoki

**Affiliations:** 1 Laboratory for Data-driven Biology, Nagoya University Graduate School of Medicine, Nagoya, Aichi 466-8550, Japan; 2 Division of Biological Science, Graduate School of Science and Technology, Nara Institute of Science and Technology, Ikoma, Nara 630-0192, Japan; 3 Life Science Collaboration Center (LiSCo), Nara Institute of Science and Technology, Takayama, Ikoma, Nara 630-0192, Japan; 4 Medilux Research Center, Nara Institute of Science and Technology, Ikoma, Nara 630-0192, Japan; 5 Graduate School of Biostudies, Kyoto University, Sakyo, Kyoto 606-8501, Japan; 6 Laboratory for Developmental Genome System, RIKEN Center for Biosystems Dynamics Research, Chuo-ku, Kobe, Hyogo 650-0047, Japan; 7 Laboratory for Data-driven Biology, Graduate School of Integrated Sciences for Life, Hiroshima University, Higashihiroshima, Hiroshima 739-8528, Japan; 8 Center for One Medicine Innovative Translational Research (COMIT), Nagoya University, Nagoya, Aichi 464-8601, Japan

**Keywords:** spatial transcriptome, single-cell RNA sequencing, zero-shot learning

## Abstract

Mutant analysis is the core of biological/pathological research, and measuring spatial transcriptomes can facilitate the understanding of the disorganized tissue phenotype. However, the high cost and technical challenges of spatial transcriptome experiments hinder the investigation of large numbers of mutants. Spatial transcriptomes have also been computationally predicted from single-cell RNA sequencing data using teaching data of spatial expression of certain genes, but the lack of teaching data for most mutants remains challenging. In various machine-learning tasks, zero-shot learning offers potential for predictions without teaching data. Here, we provided ZENomix, the zero-shot framework for predicting mutant spatial transcriptomes without teaching data (e.g., mutant spatial atlases). ZENomix accurately predicted spatial transcriptomes in Alzheimer’s model mice, Alzheimer’s human brains, and Nodal-signaling-deficient mutant zebrafish embryos. We proposed a ZENomix-based screening approach, identifying Nodal-downregulated genes in zebrafish. We expect that ZENomix offers phenotypic insights by leveraging the enormous amount of mutant/disease single-cell RNA sequencing data.

## Highlights

• ZENomix predicts mutant spatial transcriptomes in a zero-shot manner using GPLVM and MMD
• ZENomix accurately reconstructs spatial transcriptomes in disease and mutant
• Spatially informed screening identified eight Nodal-downregulated genes in zebrafish

## The bigger picture

Understanding how genes are spatially organized in tissues is essential for uncovering mechanisms of disease and developmental disorders. Spatial transcriptomics technologies allow researchers to map gene activity across tissues, but their high cost and technical complexity limit their use to a small number of conditions. By contrast, single-cell RNA sequencing has become widely adopted, producing gene expression data from hundreds of mutant and disease models. However, this approach loses spatial information when tissues are dissociated into individual cells. Existing computational methods can recover spatial information by referencing spatial gene expression atlases, but such atlases are unavailable for most mutant and disease conditions. We introduce ZENomix, a zero-shot-learning framework that predicts spatial transcriptomes of mutant or diseased tissues using only a wild-type spatial atlas as side information, without requiring any mutant-specific spatial data. This approach takes advantage of shared spatial coordinate systems between wild-type and mutant tissues. ZENomix enables researchers to identify genes with spatially disrupted expression patterns, offering improved biological insight beyond conventional differential expression analysis. We validate ZENomix across multiple species and disease contexts and demonstrate its utility for discovering genes with perturbed spatial expression. Without the need for mutant-specific spatial references, ZENomix enables spatial analysis of the vast existing single-cell RNA sequencing data from mutant and disease conditions, with potential implications for understanding disease pathology and developmental biology.

## Graphical abstract

**Figure:**
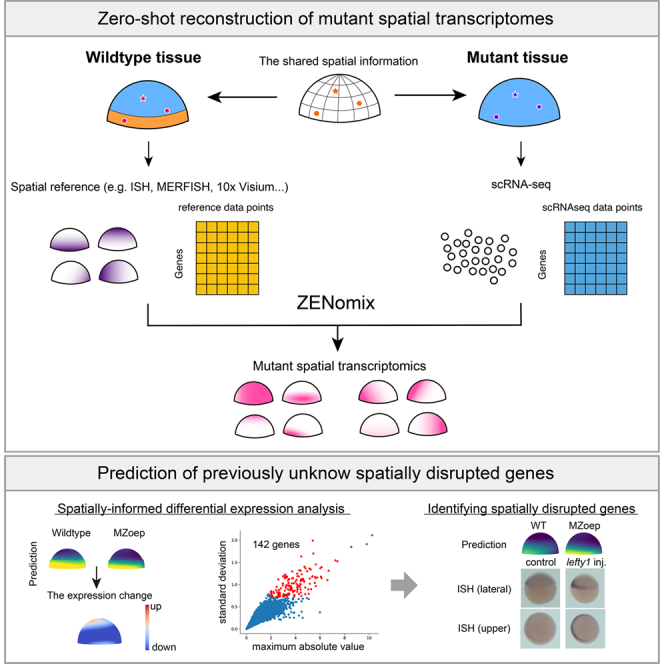

## Teaser

Measuring spatial transcriptomes in mutant tissues is costly and technically challenging. This study proposes ZENomix, a zero-shot framework that predicts mutant spatial transcriptomes without mutant-specific spatial data, using only wild-type spatial data as side information. ZENomix recovers spatial gene expression patterns across disease models and identifies previously unknown spatially disrupted genes.

## Introduction

Identifying spatial gene expression profiles is crucial for understanding whether a tissue of interest is functional in mutants and diseases. Recently developed spatially resolved transcriptomic technologies (*in situ* RNA capture for next-generation sequencing-based methods and *in situ* RNA sequencing for *in situ* hybridization [ISH]-based methods)^1^ have enabled high-throughput measurement of gene expression profiles in a spatial context, providing valuable insights into the mechanisms underlying tissue disorganization in diseases.^2^^,^^3^^,^^4^^,^^5^ However, many mutants are biologically and pathologically worth investigating. Nonetheless, comprehensive measurement of the spatial transcriptomes of these mutants is limited by the cost and technically demanding nature of the technologies.^6^ Moreover, these technologies often suffer from a trade-off between gene detection sensitivity and the number of genes measured.^7^ By contrast, methods for computationally reconstructing spatial transcriptomes from single-cell RNA sequencing (scRNA-seq) data have a high gene detection sensitivity for whole transcriptomes.^8^ According to the concept of reconstruction, dissociated scRNA-seq data are assembled by referring to teaching data of the spatial expression patterns of some genes, such as the ISH Atlas. Many methods, including our previous method, Perler, have used various algorithms to reconstruct spatial transcriptomes from scRNA-seq data.^9^^,^^10^^,^^11^^,^^12^^,^^13^^,^^14^^,^^15^^,^^16^^,^^17^^,^^18^ However, in mutant tissues, most of which have no spatial gene expression atlas, teaching data are generally unavailable, rendering the concept inapplicable.

Prediction without teaching data is a challenge for various tasks in image recognition and natural language processing that require predicting previously unknown events. However, to compensate for the lack of teaching data, existing data that do include the teaching signals of interest are trained as side information to refine the prediction. This concept, called “zero-shot learning,” has enormous potential for solving general prediction problems without teaching data, similar to how humans can predict a new event without ever experiencing it.^19^

In this study, we developed the first, to our knowledge, computational zero-shot framework (ZENomix) for the reconstruction of mutant spatial transcriptomes, without using teaching data, such as a mutant spatial reference atlas. We leveraged the wild-type spatial reference atlas in our zero-shot system, easily accessible as side information. We reasoned that although the wild type had gene expression patterns different from those of mutant tissues, the underlying spatial coordinates of tissues were comparable when the wild-type and mutant tissue morphologies were similar. The wild-type reference atlas is used as a landmark point for spatial coordinates in tissues in ZENomix, helping to add spatial information to mutant scRNA-seq data. ZENomix learns tissue spatial information by embedding a wild-type spatial reference atlas in the latent space and then mapping the mutant scRNA-seq data into this space. This latent spatial information is then used to reconstruct mutant spatial transcriptomes.

We first evaluated the performance of ZENomix in a mouse model of Alzheimer’s disease (AD) using simulated scRNA-seq data and the three human brain datasets of various spatial transcriptomics (ST) platforms (10× Visium, multiplexed error-robust fluorescence *in situ* hybridization [MERFISH],^20^ and 10× Xenium). We then used ZENomix to analyze scRNA-seq data from an early embryo of a mutant zebrafish. By comparing known ISH data for maternal-zygotic one-eyed pinhead (*MZoep*) mutants, we confirmed the spatial transcriptomes predicted by ZENomix. By predicting spatial gene expression using ZENomix, we identified previously unknown genes exhibiting spatially restricted gene expression changes, which we validated by conducting ISH experiments. These findings reveal that ZENomix provides a concept for zero-shot reconstruction of mutant spatial transcriptomes.

## Results

### Zero-shot reconstruction framework

**Figure 1:**
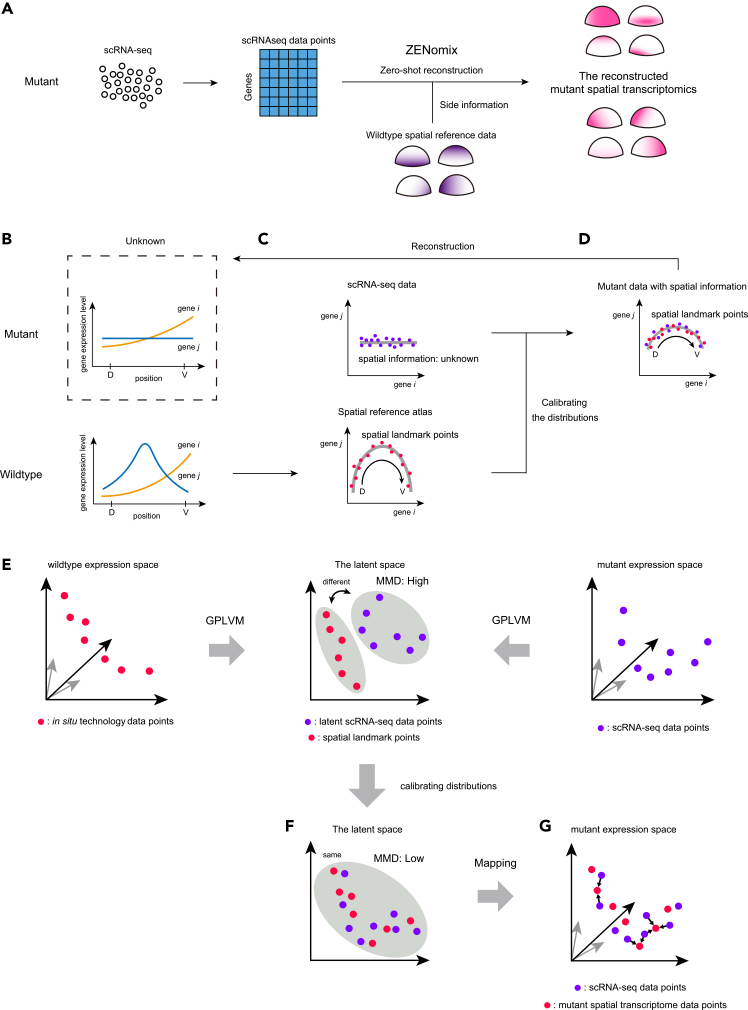
Zero-shot reconstruction concept and scheme of ZENomix (A) Reconstruction of mutant spatial transcriptomes using ZENomix. (B) Simple one-dimensional model of wild-type and mutant tissues. The model tissues exhibited varying gene expression patterns along the dorsoventral (D-V) axis. Blue and orange lines indicate the expression profiles of genes *i* and *j*, respectively. (C) Different trajectories in the gene expression space. Gene expression levels in mutants were measured using scRNA-seq, eliminating spatial information. In the wild type, gene expression levels were measured using *in situ* technology that has spatial information (black arrow) and are used as landmark points (postal codes) when adding spatial information to mutant scRNA-seq data. The red and blue points represent data from the *in situ* technology and scRNA-seq, respectively. (D) Mutant gene expression data with spatial information. The trajectories were calibrated by matching the two data point distributions. After calibrating the differences in the trajectories, the lost spatial information of the mutant scRNA-seq data points can be retrieved by referencing landmark points with spatial information. (E and F) First step of ZENomix. (E) Embedding into the latent space in ZENomix. In the wild-type expression space, there are high-dimensional *in situ* technology data points (red points, left); in the mutant expression space, there are high-dimensional scRNA-seq data points (blue points, right). ZENomix uses GPLVM to embed the wild-type data points into the latent space to obtain spatial landmark points. The mutant data points were also embedded in the latent space but with a data distribution different from that of the wild types. (F) Calibration of differences in the distributions. After mapping into the latent space, ZENomix calibrates the difference in the distributions of the two datasets by minimizing the discrepancy between the distributions (MMD; see methods). The gray-shaded region indicates the calibrated distribution. (G) Second step of ZENomix. Following the first step, the spatial landmark points derived from wild-type data are mapped onto the mutant expression space by ZENomix. Mutant spatial transcriptomes (red points) were reconstructed using the weighted average of mutant scRNA-seq data points (blue points). Black arrows indicate the weights of the scRNA-seq data points.

ZENomix is a zero-shot-learning computational method for predicting mutant spatial transcriptomes using wild-type spatial reference data as side information (Figure 1A).

Predicting mutant spatial transcriptomes without using teaching data (i.e., a mutant spatial reference atlas) is generally challenging. To better understand the ZENomix framework, we started with a simple, one-dimensional tissue with two spatial gene expression profiles (e.g., dorsoventral axis patterning of the vertebrate neural tube by Shh and BMP/Wnt^21^) (Figure 1B). The spatial information of each cell in this tissue could be expressed along its trajectory in the gene expression space (Figure 1C). Furthermore, given that wild-type spatial reference data contain gene expression and spatial information, wild-type spatial reference data points can be used as landmarks for spatial information in gene expression spaces (Figure 1C). This implies that if mutant scRNA-seq data points can be placed along this wild-type trajectory, their spatial information can be retrieved from landmark points possessing spatial information. However, in mutant tissues, the trajectory in gene expression space was distorted because of varying gene expression profiles (Figure 1C). Therefore, we calibrated the differences in these trajectories and retrieved spatial information from the mutant scRNA-seq data by comparing cell distribution in the gene expression space (Figure 1D).

Creating a zero-shot framework involves two steps: training and reconstruction. Using the abovementioned concept, the training step involves extracting the spatial information of landmark points in the tissues from the wild-type spatial reference data. In practice, spatial reference data contain tens to thousands of gene expression profiles in two- or three-dimensional tissues, for example, 47 genes in zebrafish ISH data^9^ and 12,337 genes in mouse olfactory bulb (OB) ST data.^22^ To address the high-dimensional nature of these data, ZENomix embeds wild-type spatial reference data into the latent space to obtain spatial information landmark points using the Gaussian process latent variable model (GPLVM), a nonlinear dimensionality reduction method^23^ (Figure 1E). The difference in distribution between the wild type and mutants was calibrated by minimizing the distance between the two data distributions (Figure 1F) using the maximum mean discrepancy (MMD) statistic.^24^ The second step, reconstruction, was used to obtain the mutant spatial gene expression profiles. ZENomix mapped the landmark points derived from the wild-type data back to the mutant scRNA-seq space, and the mutant spatial transcriptomes were reconstructed as the weighted average of the mutant scRNA-seq data points using Gaussian process regression (arrows in Figure 1G).

To estimate the parameters of ZENomix, we proposed a new inference scheme (vGPLVM-MMD) by merging the variational GPLVM^25^^,^^26^ and MMD statistics (see methods). In vGPLVM-MMD, ZENomix jointly optimizes a vGPLVM objective for each dataset together with an MMD-based regularization term that minimizes the discrepancy between the latent distributions of wild-type and mutant data points. Because the two datasets are unpaired, direct correspondence-based alignment, such as canonical correlation analysis (CCA),^27^ is not possible. Instead, MMD enforces distribution-level alignment in the latent space, allowing spatial landmark points learned from the wild-type data to be transferred to the mutant scRNA-seq space in a zero-shot manner. This approach learns latent variables for each dataset while encouraging a shared latent geometry, a property that is essential for cross-genotype spatial reconstruction.

### ZENomix performance on simulated data

**Figure 2:**
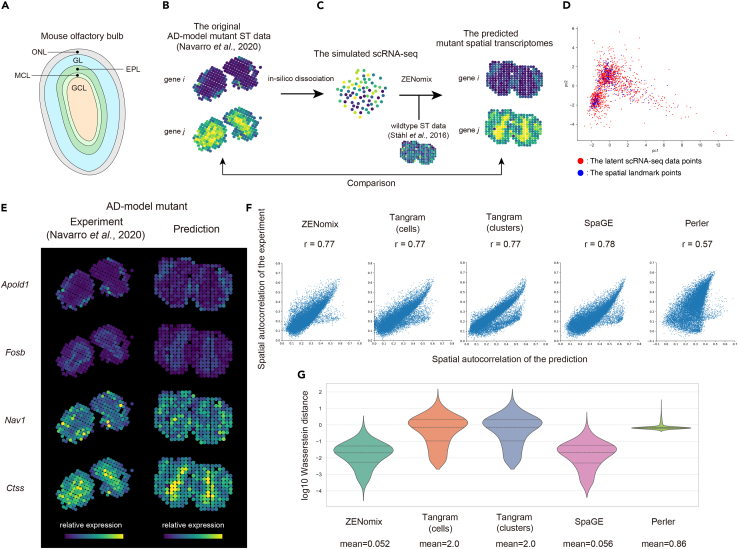
Zero-shot reconstruction of AD-mutant spatial transcriptomes (A) Anatomy of the mouse olfactory bulb.^28^ GCL, MCL, EPL, GL, and ONL indicate granular, mitral, external plexiform, glomerular, and olfactory nerve layers, respectively. (B and C) The experimental flow schematics. (B) Alzheimer’s disease mutant mouse olfactory ST data obtained from Navarro et al.^3^*In silico* dissociation of the AD-mutant ST data yielded simulated scRNA-seq data. (C) Zero-shot reconstruction using ZENomix of AD-mutant spatial transcriptomes from Navarro et al. simulated AD-mutant scRNA-seq data.^3^ Wild-type ISH data from Ståhl et al.^22^ were used as side information. Prediction accuracy can be evaluated by comparing the predicted and original AD-mutant spatial transcriptomes. (D) Scatterplot of the calibrated spatial landmark point and scRNA-seq data point distributions (Figure 2B). Principal-component analysis was used to visualize the latent space. (E) Predicted spatial transcriptomes of the AD-mutant mouse olfactory bulb. (F and G) Performance comparison across multiple methods (ZENomix, Tangram [cells mode], Tangram [clusters mode], SpaGE, and Perler) using (F) Moran’s I and (G) Wasserstein distance. (F) Scatterplots showing the spatial autocorrelation of the original and predicted AD-mutant spatial transcriptomes. Each dot indicates a gene (*n* = 16,037). (G) The violin plot represents the full distribution of gene-wise Wasserstein distances between the original and predicted AD-mutant spatial transcriptomes. Horizontal lines within the violin correspond to the 25th, 50th (median), and 75th percentiles.

To determine whether our zero-shot reconstruction framework performed well, we used simulated scRNA-seq data from the triple-transgenic AD (AD-mutant) mouse OB to evaluate ZENomix performance (Figure 2A). These ST data represent gene expression in the mouse OB, obtained at points spatially arranged in a lattice (Figure 2B). By concealing the actual spatial coordinates of the AD-mutant mouse OB data from Navarro et al., simulated scRNA-seq data (1,409 data points) were generated.^3^ The validity of this simulated scRNA-seq was confirmed by unsupervised clustering, which revealed that although a few clusters exhibited mixed cell-type characteristics (e.g., clusters containing both astrocytes and neurons), five out of the eight clusters corresponded to a single cell type, including neurons or glia (Figure S1). The ST data of the wild types from Ståhl et al.^22^ were used as spatial reference data (Figure 2C). We used the ground-truth spatial coordinates of the simulated mutant scRNA-seq data as benchmarks.

**Figure 3:**
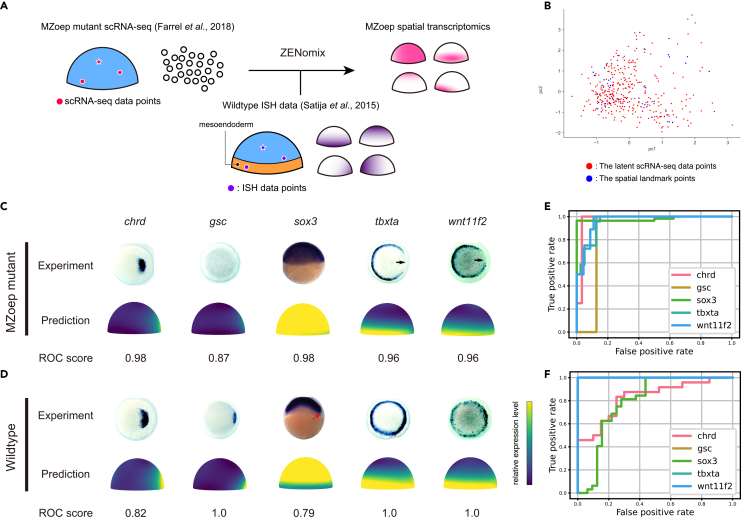
Spatial transcriptome prediction of zebrafish mutant embryos by ZENomix (A) Experimental flow schematic. ZENomix predicts the *MZoep*-mutant spatial transcriptomes from the mutant scRNA-seq data from Farrel et al.^34^ The wild-type ISH data from Satija et al.^9^ are used as side information. (B) Scatterplot of the calibrated spatial landmark point and scRNA-seq data point distributions (corresponding to Figure 2B). Principal-component analysis was used to visualize the latent space. (C and D) The experiment and prediction of the spatial transcriptomes of the (C) *MZoep* mutant and (D) wild type. Prediction performance was evaluated using the receiver operating characteristic (ROC) curve with an area under the curve (AUC). The color bar is shared between the *MZoep* mutant and the wild type. (E and F) ROC curves of the genes shown in (C) and (D) of the (E) *MZoep* mutant and (F) wild type are presented. For comparison, the ISH images of embryos at similar developmental stages were adapted, with permission, from Gritsman et al.^33^ (*chrd*, *gsc*, *tbxta*, and *wnt11f2*; top view) and Bennett et al.^37^ (*sox3*; lateral view) (license numbers: 6175390345906 and 6175390696756). The black and red arrows in the *tbxta*, *wnt11f2*, and *sox3* images were present in the original publications.

We first confirmed that ZENomix could calibrate the distributions of two latent-space data points (Figures 2D and S2A) and that the model parameters and MMD values were converged (Figure S3; Data S1). We confirmed that the reconstructed mutant spatial transcriptome was well matched to the mutant scRNA-seq data rather than the wild-type scRNA-seq data in the uniform manifold approximation and projection (UMAP) embedding (Figure S4A). We showed that ZENomix successfully predicted the spatial gene expressions of the AD-mutant mouse OB (Figure 2E). We evaluated predictive uncertainty, as ZENomix is based on Gaussian process regression (see methods), showing that these genes are predicted with high confidence (Figure S5). We then performed the same analysis using simulated wild-type scRNA-seq data from the wild-type ST data of Navarro et al. (Figure S6) To assess the predictive accuracy of ZENomix for AD-mutant, we compared the predicted spatial transcriptome with the original ST data by computing the Moran’s I statistic, a measure of spatial autocorrelation, for all genes (*n* = 16,037 genes) and found that the predicted and original spatial transcriptomes were correlated (r = 0.77; Figure 2F). Although we observed genes with low correlation between predicted and original spatial autocorrelation, pathway enrichment and Gene Ontology (GO) analyses using Enrichr^29^ indicated that these genes were not associated with known AD-related biological processes (Figure S7), suggesting that they do not represent AD-relevant spatial perturbations. To benchmark the performance of ZENomix, we compared its predictive accuracy with that of Perler^16^ (r = 0.57), Tangram^17^ (r = 0.77 in cells mode; r = 0.77 in clusters mode), and SpaGE^15^ (r = 0.78) (Figures 3F and S6) and found that ZENomix exhibited higher predictive accuracy than Perler and comparable performance to Tangram and SpaGE in terms of spatial autocorrelation. In addition to Moran’s I, we evaluated the model performance using gene-wise Wasserstein distance as a metric for the difference in the overall distribution of gene expression levels. ZENomix’s prediction showed a lower Wasserstein distance (d = 0.052) to the original spatial transcriptomes than Tangram (d = 2.0 for cells mode; d = 2.0 for clusters mode) and Perler (d = 0.86) and was comparable to that of SpaGE (d = 0.056). Combining the results from spatial autocorrelation and Wasserstein distance, ZENomix showed superior performance to Tangram and Perler. While all methods other than ZENomix are designed to integrate scRNA-seq data with spatial reference data derived from the same biological context, SpaGE achieved performance close to ZENomix despite lacking an explicit mechanism to model cross-genotype distributional shifts. These findings validated the ability of ZENomix to execute zero-shot learning to reconstruct mutant spatial transcriptomes.

### Performance benchmarking and sensitivity analysis using human brain datasets

We further benchmarked ZENomix against Tangram and SpaGE using three human brain datasets generated across multiple ST platforms with varying spatial resolutions (PFC_visium [PFC, prefrontal cortex],^30^^,^^31^^,^^32^ MTG_merfish [MTG, middle temporal gyrus],^30^ and PFC_xenium^30^). Performance was evaluated using Moran’s I and gene-wise Wasserstein distance in cross-genotype settings and spot-wise Spearman’s correlation in same-genotype settings. In the PFC_visium and PFC_xenium datasets, ZENomix achieved higher Spearman’s correlation than Tangram (cells mode) and SpaGE and performance comparable to Tangram (clusters mode), whereas it relies on averaged cell-type expression profiles (Figure S8). In the MTG_merfish dataset, SpaGE achieved the highest Spearman’s correlation, while ZENomix and Tangram showed comparable performance (Figure S8). Across all datasets, ZENomix and SpaGE showed similar performance in terms of Moran’s I and Wasserstein distance, whereas Tangram exhibited inconsistent behavior across these metrics (Figure S9). Overall, these results indicate that ZENomix performed robustly across platforms and achieved performance comparable to existing methods, while it exceeded them on sequencing-based ST platforms in particular.

We conducted sensitivity analyses of key parameters, including the number of landmark genes, the latent space dimensionality, MMD kernel bandwidth, and the number of inducing points, indicating that the number of inducing points and the latent-space dimensionality are important for the performance of ZENomix (Figure S10). In addition, we systematically evaluated how ZENomix’s performance varies with the number of landmark genes and the level of scRNA-seq sparsity by introducing controlled dropouts (Figure S11). Furthermore, we confirmed that ZENomix’s performance is robust in terms of scRNA-seq batch effects (Figures S12 and S13). Lastly, the runtime benchmark showed that ZENomix could successfully process scRNA-seq datasets with up to 100,000 cells using up to 48 GB of GPU memory, demonstrating its practical scalability on commonly available GPUs (Figure S14).

### Predicted spatial transcriptomes in zebrafish mutant

We used ZENomix to analyze the scRNA-seq data from mutant zebrafish embryos, for which the previous experimental method was challenging to apply due to the small, dome-shaped tissue. We used scRNA-seq data from an early embryo (at the 50% epiboly stage) of an *MZoep*^33^ mutant obtained by Farrel et al.^34^ The *MZoep* mutant lacks a Nodal signaling co-receptor, resulting in defects in the dorsal organizer, inducing mesoendodermal formation^35^^,^^36^ (Figure 3A). For the spatial reference data, we used binary ISH data, including 47 genes obtained by Satija et al.^9^

First, we confirmed that ZENomix calibrated the distribution discrepancies between the two data points in latent space (Figures 3B and S2; Data S1). Then, we confirmed that the reconstructed mutant spatial transcriptome was well matched to the mutant scRNA-seq data rather than the wild-type scRNA-seq data in the UMAP embedding (Figure S4B). The spatial transcriptomes of several genes known to be altered in *MZoep* mutants were predicted using ZENomix,^33^^,^^37^ and all predictions were consistent with those in the previously published ISH images (Figures 3C–3F). The predictive uncertainty revealed that expression of these genes was predicted with high confidence (Figure S5).

To quantify the predictions, the ground-truth binary ISH data for MZoep embryos were manually generated (Figure S15 and Data S2). We conducted the same analysis using other methods (Figure S16). Thus, ZENomix recapitulates the spatial gene expression changes caused by Nodal signaling mutations. These findings clearly indicate that ZENomix might successfully predict mutant spatial transcriptomes in a zero-shot manner.

### Identifying spatially differentially expressed genes

**Figure 4:**
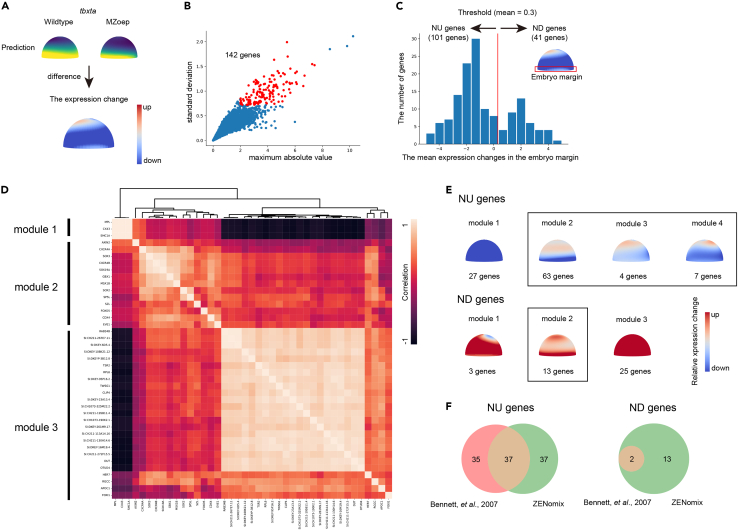
Screening of the putative spatially differentially expressed genes (A) Schematic depicting the expression changes calculated by subtracting the predicted spatial transcriptome of the wild-type from that of the *MZoep*-mutant embryos. (B) Gene screening scatterplot. The *x* and *y* axes indicate the maximum absolute value and standard deviation of the expression changes, respectively. Each dot indicates a gene (*n* = 26,545 genes). The red dots indicate selected genes (nodal-associated genes; *n* = 142). (C) Histogram of mean expression changes within the embryo margin. The red line (mean = 0.3) indicates the classification threshold between NU and ND genes. The red box in the inset indicates the embryo margin, within which the mean values were calculated. (D) Hierarchical clustering of putative ND genes. The heatmap indicates the correlation between gene expression changes among newly screened ND genes. (E) The average spatial gene expression changes in each module. Four and three modules for NU and ND genes are shown. The black rectangles indicate the modules included in further analysis. (F) Venn diagrams for sets of putative NU and ND genes screened by ZENomix and from a previous study by Bennett and colleagues.^37^

To identify the spatially differentially expressed (DE) genes in *MZoep* embryos, we compared the reconstructed mutant spatial transcriptome data with those of the wild type (Figure 4). First, we computed the difference between the reconstructed spatial transcriptomes in the wild type and mutant (Figure 4A). We then plotted the maximum absolute value and standard deviation of the expression changes in the *MZoep*-mutant transcriptome for each gene (*n* = 26,545 genes) and screened 142 Nodal-associated genes (Figure 4B). We focused on expression changes for further screening in the embryo margin (red box in Figure 4C), mostly affected by Nodal signaling defects. By plotting the average expression changes only in the embryo margin, the Nodal-associated genes were classified into two groups: 101 and 41 putative Nodal-upregulated (NU) and putative Nodal-downregulated (ND) genes, respectively (Figures 4C and S17–S19).

We then performed hierarchical clustering for each putative NU and ND gene to better understand Nodal-associated gene expression alterations (Figure 4D). We identified four and three modules in putative NU and ND genes, respectively (Figure 4E; Table S1). Finally, we excluded the genes in module 1 of the putative NU genes and modules 1 and 3 of the putative ND genes, as they showed universal gene expression changes in the whole embryo, yielding 87 putative spatially DE genes (74 putative NU genes and 13 putative ND genes). Notably, 50.0% (37/74) of these putative NU genes and 15.3% (2/13) of these putative ND genes were shared with 72 and 2 genes, respectively, identified in a similar bulk microarray screening in *MZoep* embryos,^37^ suggesting that our screening was consistent with that of a previous study (Figure 4F).

### New genes repressed by nodal signaling

Nodal signaling is critical to the induction and maintenance of the dorsal organizer and repression of ectodermal cell fates.^37^ Bennett et al. suggested that Nodal represses some target genes; nonetheless, only two genes (*sox2* and *sox3*) have been identified as downregulated via Nodal signaling.^37^ Notably, ZENomix screening revealed 13 putative ND genes, including *sox2*, *sox3*, and 11 unknown genes (Figure 4F). To assess the statistical significance of these candidate genes, we employed a *t* test with Benjamini-Hochberg (BH) correction (Figures S20A and S20B). We confirmed that significantly higher expression (SHE) genes overlapped with 11 of the 13 identified ND genes. We compared ZENomix-based spatial DE results with baseline differential expression analysis performed directly on scRNA-seq data, as well as spatially DE analyses derived from Tangram and SpaGE reconstructions. While a comparable fraction of the ND genes was recovered using SpaGE-based spatial predictions, Tangram-based predictions and conventional scRNA-seq DE analysis detected only a single ND gene (Figure S21; Data S3), highlighting the added value of spatially DE analysis. We then performed pathway enrichment and GO analyses on 13 putative ND genes using Enrichr^29^ (Figures S20C–S20H; Data S4). We found significant enrichment for processes related to Nodal signaling and mesoendodermal formation in significantly lower expression (SLE) genes, confirming that these gene lists are related to Nodal signaling. Notably, ectodermal and neurogenic development processes were significantly enriched in the identified ND genes. These functional categories are consistent with the known roles of Nodal signaling, strengthening the biological relevance of our findings.

**Figure 5:**
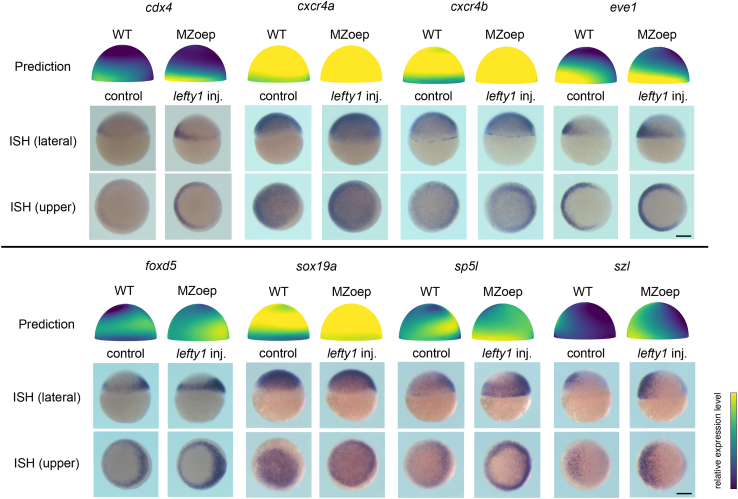
ISH validation of the nodal-downregulated genes discovered from ZENomix-predicted mutant spatial transcriptomes Whole-mount ISH experiments on eight identified ND genes. Top to bottom: ZENomix-predicted expression pattern, lateral ISH view, and top ISH view. For each gene, wild-type and mutant expression patterns are displayed (ZENomix prediction: wild-type and *MZoep*-mutant embryos; ISH experiment: control and *lefty1*-injected embryos). Scale bar, 200 μm.

To validate the candidate ND genes, we used ISH to assess whether the reconstructed spatial transcriptomes of the 11 unknown genes in *MZoep*-mutant embryos followed the spatial gene expression profiles determined by the ISH experiments. Since *MZoep* is not kept in our zebrafish facility, and the *lefty1* overexpression embryo, which encodes the Nodal inhibitor, can serve as a widely used phenocopy of *MZoep* mutants and *cyclops/squint* double mutants,^38^^,^^39^ we used *lefty1*-overexpressed embryos instead of *MZoep* mutants for ISH evaluation of ZENomix predictions (*lefty1*-overexpressed embryos; see methods). We found that among the 11 candidate genes, the predicted spatial gene expression patterns of eight genes (*cdx4*, *cxcr4a*, *cxcr4b*, *eve1*, *foxd5*, *sox19a*, *sp5l*, and *szl*) correlated with the ISH results, indicating that ZENomix discovered eight new genes repressed via Nodal signaling (Figure 5).

The expression patterns of the other three candidate genes (*axin2*, *gbx1*, and *msx1b*) were inconsistent with those of the ISH results (Figure S22A). In the ISH assays, *axin2* specifically showed comparable ubiquitous expression patterns between wild-type and *lefty1*-injected embryos, but the number of *axin2*-positive cells increased in *MZoep*-mutant embryos compared to wild-type embryos in the scRNA-seq data (Figure S22B). In contrast, *gbx1* and *msx1b* were unexpressed in either embryo in the ISH experiments, despite being expressed in the scRNA-seq data (Figure S22C). These discrepancies may reflect a combination of technical differences between scRNA-seq and ISH, as well as residual biological differences between *MZoep*-mutant and *lefty1* overexpression embryos. To our knowledge, transcriptome-wide comparisons between *MZoep*-mutant and *lefty1* overexpression embryos have not been reported, making it difficult to quantitatively assess the relative contributions of these factors.

### ZENomix limitations

We used ZENomix on *bcd*-knockdown (KD) *D*. *melanogaster* embryos to test its predictability in mutants with a tissue structure rearrangement. During *Drosophila* development, the anterior-posterior (A-P) axis was created by morphogen gradients of the anterior *bcd* and posterior *nanos* genes. In contrast, in *bcd*-KD embryos, the anterior identity of the embryo was converted to a posterior identity, resulting in a reorganization of embryonic structural allocation, with loss of the head and thorax^40^ (Figure S23A).

First, we attempted to reconstruct *bcd*-KD spatial transcriptomes in a zero-shot manner (Figures S23B and S23D). In this experiment, *bcd*-KD scRNA-seq data from Sakaguchi et al.^41^ and wild-type fluorescence ISH (FISH) data (84 genes) from the Berkeley Drosophila Transcription Network Project (BDTNP)^42^^,^^43^^,^^44^^,^^45^ were used. We observed that the reconstruction of the *bcd-*KD spatial transcriptomes failed completely. As a control experiment, we used ZENomix to reconstruct *bcd*-KD spatial transcriptomes using the teaching data and *bcd*-KD ISH from Staller et al.^40^ (13 genes) (Figures S23C and S23D). Under these conditions, we confirmed that ZENomix successfully reconstructed *bcd*-KD spatial transcriptomes. To investigate why ZENomix failed under zero-shot conditions, we compared *bcd*-KD spatial transcriptomes with and without teaching data by estimating the original location of the scRNA-seq data point (see methods). In the reconstruction with teaching data (i.e., *bcd*-KD ISH data), the scRNA-seq data points were equally estimated along the A-P axis, whereas most scRNA-seq data points were estimated to be biased toward the posterior region in the reconstruction without teaching data (i.e., wild-type ISH data) (Figures S23E–S23G). These findings indicate that ZENomix incorrectly assigned the origin of the scRNA-seq data points obtained from the anterior region to the posterior region due to A-P identity conversion.

The A-P axis of *bcd*-KD embryos was folded in half in the gene expression space, whereas the A-P axis of wild-type embryos was clearly distinguished. We reasoned that the A-P conversion of *bcd*-KD embryos could explain the failure of origin estimation (Figure S23H). Thus, ZENomix does not reliably predict spatial transcriptomes when the spatial trajectories of wild-type and mutant genes in the gene expression space are not correctly calibrated.

## Discussion

We developed ZENomix, a computational framework for reconstructing mutant spatial transcriptomes without teaching data, by introducing the zero-shot reconstruction concept. ZENomix recovers spatial information from mutant scRNA-seq data by using the wild-type spatial gene expression atlas as side information and extracting landmark points of spatial coordinates in tissues from the wild-type spatial reference atlas. Using simulated and real scRNA-seq data, we showed that ZENomix reliably predicted the spatial transcriptomes of an AD-mutant mouse model, the AD human brain cortex, and *MZoep*-mutant zebrafish embryos. Furthermore, using this spatially informed screening approach based on ZENomix predictions, we discovered eight ND genes in early zebrafish embryos. Therefore, ZENomix is useful for identifying genes with perturbed expression and can provide new insights into the mutant or diseased tissue pathogenesis.

ZENomix does not require identical tissue morphology or cell-type composition between mutant scRNA-seq data and wild-type spatial reference atlas. Instead, ZENomix requires that the spatial coordinate (e.g., A-P axis) of the tissue be preserved. For example, even in *MZoep* zebrafish embryos, epiboly defects caused by the lack of Nodal signaling introduce slight structural and cell state differences in *MZoep*-mutant embryos.^34^^,^^46^ Nevertheless, the animal-vegetal axis is retained across the two genotypes, enabling successful zero-shot reconstruction with ZENomix. Similarly, in the mouse OB, the laminar organization is preserved, which supports accurate reconstruction. By contrast, ZENomix may be less effective in scenarios where the underlying spatial coordinate system is severely disrupted or when novel spatial domains emerge in mutant tissues. For example, ZENomix failed to reconstruct the spatial transcriptomes of *bcd*-KD *Drosophila* embryos, in which the A-P axis is converted from wild-type embryos. These cases represent an important limitation of the framework and define conditions under which experimental spatial profiling would be required. This coordinate-preservation hypothesis also provides practical guidance for selecting an appropriate wild-type spatial reference. Although the reference does not need to match the mutant tissue in morphology, developmental stage, or experimental protocol, it should preserve the same global spatial coordinates that serve as transferable spatial landmarks. In practice, spatial references that are incomplete or lack coverage of the relevant spatial axes are expected to be less suitable. ZENomix provides gene-wise predictive variance from the Gaussian process regression as a credibility score. Regions with high predictive covariance should be treated as low-confidence mappings that may reflect extrapolation to coordinates where no cells exist in the mutant tissue rather than biologically meaningful spatial patterns such as fate or identity changes in cells that still physically occupy the corresponding coordinates.

The proposed zero-shot reconstruction framework complements existing spatial transcriptomic and data integration approaches by enabling spatial analysis in settings where generating spatial data is impractical. First, although spatial transcriptomic technologies are becoming increasingly accessible, generating spatial data for every mutant remains costly and labor-intensive. This limitation is particularly relevant for exploratory studies or multi-condition experimental designs, where the biological significance of individual mutants has not yet been established. In such cases, ZENomix enables *in silico* spatial analysis of mutant or disease scRNA-seq data, facilitating the identification of promising mutants for follow-up spatial experiments. Second, existing methods to integrate scRNA-seq and ST require spatial transcriptomic data from the same genotype or disease condition. In contrast, ZENomix operates in a zero-shot manner by leveraging only a wild-type spatial reference to infer spatial gene expression changes in mutant or disease tissues. This feature is particularly valuable for human datasets, in which matched spatial profiling of patient samples is often unavailable. Beyond spatial reconstruction, ZENomix also enables spatially DE analysis, revealing genes with perturbed spatial patterns that cannot be identified from scRNA-seq alone. Overall, ZENomix is a complementary framework that extends its utility for hypothesis generation and data reuse when direct spatial profiling is limited.

Although many methods using scRNA-seq data have been used to rebuild spatial transcriptomes,^9^^,^^10^^,^^11^^,^^12^^,^^13^^,^^14^^,^^16^^,^^17^^,^^18^ none is designed to achieve the zero-shot reconstruction of mutant spatial transcriptomes for two reasons. First, the scRNA-seq and *in situ* data are modeled assuming they were obtained under the same biological conditions. For example, the neural-network-based method gimVI^14^ assumes that *in situ* and scRNA-seq data are generated from a shared latent biological state using a similar function. ZENomix overcomes this bottleneck by modeling mutant and wild-type data using different functions based on Gaussian process priors. In our benchmarks, SpaGE achieved performance comparable to ZENomix despite lacking an explicit mechanism to model cross-genotype distributional shifts. This result suggests that SpaGE’s domain-adaptive feature projection^47^ can provide implicit robustness to distributional differences across genotypes. In contrast, ZENomix provides a principled generative framework in which genotype difference is explicitly formulated, enabling zero-shot spatial reconstruction. Second, most previous methods optimized similarity measures (e.g., cosine similarity,^17^ mean squared error,^18^ inverse correlation,^13^ and mutual information^16^) between *in situ* data and reconstructed transcriptomes, making integration of various genotyped data unfeasible. By contrast, ZENomix relies on Bayesian estimation of spatial transcriptomes, enabling reconstruction without any similarity measures.

Our newly developed screening approach, based on ZENomix predictions, is critical for identifying genes whose expression is perturbed in mutant tissues. Our screening approach can use data related to gene expression changes in specific regions between the wild type and the mutant (e.g., the embryonic margin in *MZoep*), in contrast to bulk transcriptomics-based screening using microarrays and RNA-seq. Using our spatially informed screening method, we discovered eight previously unknown, new ND genes. We also identified 74 putative NU genes, consistent with the previous bulk microarray-based screening.^37^ These findings highlight the significance of spatial information in KD/knockout analyses, suggesting that ZENomix can identify new biological mechanisms through zero-shot reconstruction.

This study has several limitations that point to directions for future methodological development. While ZENomix assumes that a global spatial coordinate system is preserved between wild-type and mutant tissues, it does not currently model changes in tissue morphology explicitly. Developing computational frameworks that jointly model gene expression and tissue deformation could extend the applicability of ZENomix to mutants exhibiting more pronounced morphological alterations. In addition, ZENomix assigns mutant scRNA-seq data points to spatial locations solely based on gene expression profiles. Incorporating prior biological information, such as gene regulatory networks or lineage relationships, may further refine spatial inference in challenging settings.

## Methods

### Zebrafish

The wild-type strain RIKEN WT (RW) was used in this study. All zebrafish experiments were approved by the animal studies committee of the Nara Institute of Science and Technology.

### Whole-mount ISH

Whole-mount ISH was conducted as described previously.^48^^,^^49^ Briefly, PCR was used to generate template DNAs for *axin2*, *cdx4*, *cxcr4a*, *eve1*, *foxd5*, *gbx1*, *msx1b*, *sox19a*, *sp5l*, and *szl* using forward and reverse primers with T3 and T7 sequences, respectively, inserted at the 5′ ends; the T3 and T7 primers were used to confirm the sequence. pCRII-*cxcr4b* was used as the template, and T7 or SP6 RNA polymerases were used to synthesize DIG antisense RNA probes.

### *lefty1* overexpression in zebrafish embryo

*lefty1* mRNAs were synthesized using the mMessage mMachine SP6 transcription kit (Thermo Fisher Scientific) with pCS2-*lefty1* (kindly gifted by Dr. Masashiro Hibi) as the template.^39^ Then, 5 pg of the synthesized *lefty1* mRNA was injected into one-cell-stage zebrafish embryos, and the embryos were used for ISH.

### ZENomix model

ZENomix uses wild-type spatial reference data and mutant scRNA-seq data as inputs. In spatial reference data (e.g., ST data) generated using *in situ* methods, gene expression vectors are available for all regions/cells whose locations in the tissue are known. The gene expression vector of gene *j* is represented as $y_{j}^{\left(W\right)}=\left(y_{j,1}^{\left(W\right)},\ldots ,y_{j,n_{W}}^{\left(W\right)},\ldots ,y_{j,N_{W}}^{\left(W\right)}\right)\in R^{N_{W}}$, where cells are indexed by *n*_*W*_ (*n*_*W*_ ∈ {1, 2, *…*, *N*_*W*_}), and *N*_*W*_ is the total number of cells in the tissue of interest. By contrast, in the scRNA-seq dataset of the genotype of interest, gene expression vectors lack information regarding the location of cells in the tissue. The expression vector of gene *j* is represented as $y_{j}^{\left(M\right)}=\left(y_{j,1}^{\left(M\right)},\ldots ,y_{j,n_{M}}^{\left(M\right)},\ldots ,y_{j,N_{M}}^{\left(M\right)}\right)\in R^{N_{M}}$, where cells are indexed by *n*_*M*_ (*n*_*M*_ ∈ {1, 2, *…*, *N*_*M*_}), and *N*_*M*_ is the total number of cells used for scRNA-seq measurement. The number of dimensions of these two data points, *p*, is the same because we considered landmark genes.

#### Generative model

The scRNA-seq and spatial reference data were modeled as a generative model:

(Equation 1) P(X)=N(X|0,I)and

(Equation 2) $$P\left(Y^{\left(W\right)},Y^{\left(M\right)}|X,\sigma^{2}\right)=\Pi_{j}P\left(y_{j}^{\left(W\right)}|X,\sigma^{2}\right)P\left(y_{j}^{\left(M\right)}|X,\sigma^{2}\right)\text{,}$$

where *N*(0,*I*) represents a standard Gaussian distribution with *q* dimensions; $Y^{\left(W\right)}\in R^{{p\times N}_{W}}$ and $Y^{\left(M\right)}\in R^{{p\times N}_{M}}$ indicate *in situ* and scRNA-seq data matrices, respectively; *X* indicates the common latent variable matrix (spatial landmark points) with dimension *q*; and *h* indicates the genotype or modality of data (*h* ∈ {W (wild-type spatial reference data), M (mutant scRNA-seq data)}). The following functions of low-dimensional latent variables were used to generate gene expression:

(Equation 3) $$y_{j}^{\left(h\right)}=f_{j}^{\left(h\right)}\left(X\right)+\epsilon \text{,}$$

where *ϵ* indicates the Gaussian observation noise with zero mean and standard deviation *σ*. Note that *σ* is common among the data modalities. The aim was to estimate the posterior distribution of the latent variables, denoted as *P*(*X*|*Y*^(*W*)^,*Y*^(*M*)^).

To assume a nonlinear transformation from latent variables to gene expression levels, the Gaussian process prior was placed on the projection function $f_{j}^{\left(h\right)}$ as follows:

(Equation 4) $$P\left(y_{j}^{h}|X,\sigma \right)=N\left(0,K_{h}+\sigma^{2}I\right)\text{,}$$

where *K*_*h*_ is the gram matrix defined by the kernel function *k*(*x*^(*h*)^,*x*′^(*h*)^;θ_*k*_) between two distinct latent variables, *x*^(*h*)^ and *x*′^(*h*)^ with kernel hyperparameters, *θ*_*k*_, and *x*^(*h*)^ is a latent variable corresponding to each observation, *y*^(*h*)^.

#### Inference scheme

The ZENomix model was established to estimate the posterior probabilities of shared latent variables (spatial landmark points) *P*(*X*|*Y*^(*W*)^,*Y*^(*M*)^). However, because the two datasets are unpaired, it was difficult to estimate the latent variables shared by *Y*^(*W*)^ and *Y*^(*M*)^, implying that we cannot use the methods for paired data (e.g., CCA^27^ or multimodal mixture-of-expert variational autoencoders^50^). To solve this, vGPLVM-MMD, a new inference scheme that extracts spatial landmark points from wild-type data, was proposed.

The vGPLVM-MMD scheme comprises two parts: the first part independently maps *Y*^(*W*)^ and *Y*^(*M*)^ onto a low-dimensional latent space and the second part matches the latent data distributions. In the first part, the generative model is separated into two distinct models, *Y*^(*W*)^ and *Y*^(*M*)^, where *X*^(*h*)^ is a separate latent variable matrix corresponding to each data matrix *Y*^(*h*)^. Consequently, gene expression is generated by the function of the low-dimensional latent variable *X*^(*h*)^, which varies for each data point, as shown below:

(Equation 5) $$P\left(X^{\left(h\right)}\right)=N\left(X^{\left(h\right)}|0,I\right)\text{,}$$

(Equation 6) $$P\left(Y^{\left(h\right)}|X^{\left(h\right)},\sigma^{2}\right)=\Pi_{j}P\left(y_{j}^{\left(h\right)}|X^{\left(h\right)},\sigma^{2}\right),\text{and}$$

(Equation 7) $$y_{j}^{\left(h\right)}=f_{j}^{\left(h\right)}\left(X\right)+\epsilon \text{,}$$

where the Gaussian process prior is placed on the projection function $f_{j}^{\left(h\right)}$. From this model, we can estimate the posterior distributions *P*(*X*^(*h*)^|*Y*^(*h*)^) for each data point. In practice, we used variational inference to estimate the approximated posterior distribution *q*(*X*^(*h*)^) rather than *P*(*X*^(*h*)^|*Y*^(*h*)^). *q*(*X*^(*W*)^) and *q*(*X*^(*M*)^) exhibit different distributions in this model.

This model (Equations 5, 6, and 7) is known as the Bayesian GPLVM.^25^ As the posterior distribution is analytically intractable in this model, Titsias and Lawrence^25^ and Damianou et al.^26^ developed a vGPLVM using inducing inputs. Following their methods, the generative model can be described as follows:

(Equation 8) $$P\left(X^{\left(h\right)}\right)=\Pi_{n_{h}}N\left(x_{n_{h}}^{\left(h\right)}|0,I\right)\text{,}$$

(Equation 9) $$P\left(U|X_{u}\right)=\Pi_{j}N\left(u_{j}|0,K_{uu}\right)\text{,}$$

(Equation 10) $$P\left(F^{\left(h\right)}|U,X^{\left(h\right)},X_{u}\right)=\Pi_{j}N\left({f_{j}}^{\left(h\right)}|K_{hu}{K_{uu}}^{-1}u_{j},Tr\left(K_{h}\right)-K_{hu}{K_{uu}}^{-1}K_{uh}\right),\text{and}$$

(Equation 11) $$P\left(Y^{\left(h\right)}|F^{\left(h\right)}\right)=\Pi_{j}N\left(y_{j}^{\left(h\right)}|{f_{j}}^{\left(h\right)},\sigma^{2}I\right)\text{,}$$

where $X_{u}\left(=\left(x_{1}^{\left(u\right)}\ldots x_{m}^{\left(u\right)}\ldots x_{N_{u}}^{\left(u\right)}\right)\right)\in R^{{q\times N}_{u}}$ and $U\;\left(={\left(u_{1\;}\;\ldots u_{j\;}\;\ldots \;u_{p\;}\right)}^{T}\right)\in R^{p\times N_{u}}$ indicate the induced inputs in the latent space and extra samples in the observation space, respectively, and *F*^(*h*)^ indicates the GP mapping values. In Equation 10, the gram matrices $K_{hu}\in R^{N_{h}\times N_{u}}$ and $K_{uu}\in R^{N_{u}\times N_{u}}$ are defined as follows:

(Equation 12) $${\left(K_{hu}\right)}_{nm}=k\left(x_{n}^{\left(h\right)},x_{m}^{\left(u\right)}\right)\;\text{and}$$

(Equation 13) $${\left(K_{uu}\right)}_{mm^{\prime }}=k\left(x_{m}^{\left(u\right)},x_{m^{\prime }}^{\left(u\right)}\right)\text{.}$$

Using a Gaussian distribution, the variational inference is used to approximate the true posterior *P*(*X*^(*h*)^|*Y*^(*h*)^) as follows:

(Equation 14) $$q\left(X^{\left(h\right)}\right)=\prod_{n}^{N_{h}}N\left(x_{n}^{\left(h\right)}|\mu_{n}^{\left(h\right)},s^{2}I\right)\text{,}$$

where *s* indicates the noise intensity, which is shared by all distributions. The lower bound of *P*(*Y*^(*h*)^) is expressed as follows:

(Equation 15) $$F\left(q\left(X^{\left(h\right)}\right)\right)=\sum_{j}^{p}{\hat{F}}_{j}\left(q\left(X^{\left(h\right)}\right)\right)-KL\left(q\left(X^{\left(h\right)}\right)|p\left(X^{\left(h\right)}\right)\right)\text{,}$$

(Equation 16) $${\hat{F}}_{j}\left(q\left(X^{\left(h\right)}\right)\right)=\log\left[\frac{{\left(\beta \right)}^{\frac{N_{h}}{2}}{\left|K_{uu}\right|}^{\frac{1}{2}}}{{\left(2\pi \right)}^{\frac{N_{h}}{2}}{\left|\beta \Psi_{2}+K_{uu}\right|}^{\frac{1}{2}}}e^{-\frac{1}{2}{y_{j}}^{T}Wy_{j}}\right]-\frac{\beta \psi_{0}}{2}+\frac{\beta }{2}Tr\left({K_{uu}}^{-1}\Psi_{2}\right)\text{,}$$

(Equation 17) $$W=\beta I-\beta^{2}\Psi_{1}{\left(\beta \Psi_{2}+K_{uu}\right)}^{-1}{\Psi_{1}}^{T}\text{,}$$

(Equation 18) $$\psi_{0}=Tr\left(<K_{h}{>}_{q\left(X^{\left(h\right)}\right)}\right)\text{,}$$

(Equation 19) $$\Psi_{1}=<K_{hu}{>}_{q\left(X^{\left(h\right)}\right),}\;\text{and}$$

(Equation 20) $$\Psi_{2}=<K_{hu}{K_{hu}}^{T}{>}_{q\left(X^{\left(h\right)}\right)}\text{,}$$

where <·>_*p*(*x*)_ denotes expectation under the distribution *p*(*x*) and *KL*(*q*(*x*)‖*p*(*x*)) indicates the Kullback-Leibler (KL) divergence between distributions *q*(*x*) and *p*(*x*).

In our implementation, *Ψ* statistics (*ψ*_0_, *Ψ*_1_, and *Ψ*_2_) were computed using the Gaussian-Hermite approximation, as in Gpy.^51^ The latent representation of each data point can be obtained by maximizing the variational bound for each genotype, *h*.

In the second part of the vGPLVM-MMD scheme, the independently derived posterior distributions *q*(*X*^(*W*)^) and *q*(*X*^(*M*)^) are combined, such that the two distributions may be nearly matched as *q*(*X*^(*W*)^) ≈ *q*(*X*^(*M*)^) to extract the common latent variable *X*. To make the two distributions almost identical, the distance between the two distributions, *q*(*X*^(*W*)^) and *q*(*X*^(*M*)^), was minimized. MMD^24^ was used to compute the distance (see the subsequent MMD calculations section):

(Equation 21) $$Dist\left(q\left(X^{\left(W\right)}\right),q\left(X^{\left(M\right)}\right)\right)≔MMD\left(M^{\left(W\right)},M^{\left(M\right)}\right)\text{,}$$

where $M^{\left(h\right)}(=\left(\mu_{0}^{\left(h\right)}\;\ldots \;\mu_{n}^{\left(h\right)}\ldots \mu_{N_{h}}^{\left(h\right)}\right)$) is the mean of *q*(*X*^(*h*)^).

#### MMD calculations

MMD is the nonparametric distance between two sample distributions embedded in a reproducing kernel Hilbert space (RKHS). Let us assume a general situation where two data samples, *X* and *Y*, have identical dimensions. The empirical estimate of the MMD between the data points *X* and *Y* is as follows:

(Equation 22) $$MMD\left(X,Y\right)=\|\frac{1}{N_{x}}\sum_{n_{x}}^{N_{x}}\phi \left(x_{n_{x}}\right)-\frac{1}{N_{y}}\sum_{n_{y}}^{N_{y}}\phi \left(y_{n_{y}}\right){\|}^{2}\text{,}$$

where *ϕ* indicates the kernel-induced feature map. By expanding Equation 22 and replacing the inner products with their kernel values (the kernel trick), MMD is given as

(Equation 23) $$MMD\left(X,Y\right)=\frac{1}{{N_{x}}^{2}}\sum_{n_{x},{n_{x}}^{\prime }}l\left(x_{n_{x}},x_{{n_{x}}^{\prime }}\right)-\frac{2}{N_{x}N_{y}}\sum_{n_{x},n_{y}}l\left(x_{n_{x}},y_{n_{y}}\right)+\frac{1}{{N_{y}}^{2}}\sum_{n_{y},{n_{y}}^{\prime }}l\left(y_{n_{y}},y_{{n_{y}}^{\prime }}\right)\text{,}$$

where *l*(*x*, *y*) denotes the kernel function with MMD hyperparameters, *θ*_*l*_. When the RKHS is universal, the MMD approaches zero asymptotically if and only if the two distributions are the same.^24^ MMD hyperparameters are not optimized during posterior inference. For large datasets, we additionally employ a random Fourier feature approximation^52^^,^^53^ to reduce the cost of MMD calculation, as in GPJax.^54^

#### Variational inference

To estimate the parameters and hyperparameters of ZENomix, we integrated the first and second parts of the vGPLVM-MMD. Thus, the cost function of the vGPLVM-MMD can be denoted as follows:

(Equation 24) $$\begin{array}{l} L\left({X_{u},M}^{\left(W\right)},M^{\left(M\right)},S,{\sigma ,\theta }_{k}\right)=\\ F\left(q\left(X^{\left(W\right)}\right)\right)+F\left(q\left(X^{\left(M\right)}\right)\right)-Dist\left(q\left(X^{\left(W\right)}\right),q\left(X^{\left(M\right)}\right)\right)\text{.} \end{array}$$

This cost function was maximized using a gradient-based optimization algorithm (see the ZENomix implementation section) to extract the common latent variables of the two datasets. The MMD hyperparameters *θ*_*l*_ are fixed in ZENomix. The computational cost of evaluating *F*(*q*(*X*^(*h*)^)) is $O\left(N_{h}*{N_{u}}^{2}\right)$ , and that of *MMD*(*M*^(*W*)^,*M*^(*M*)^) is $O\left({\left(N_{I}+N_{R}\right)}^{2}\right)$. If $N_{u}=O\left(\sqrt{N_{I}+N_{R}}\right)\text{,}$ the computational complexity of evaluating *L* is *O*((*N*_*I*_ + *N*_*R*_)^2^).

#### Spatial reconstruction

In the second step of ZENomix, the mutant spatial gene expression profiles of gene *j* are obtained by mapping the latent variables of wild-type *in situ* data, *X*^(*W*)^, to mutant scRNA-seq space as $F_{j}^{*\left(M\right)}≔f_{j}^{\left(M\right)}\left(X^{\left(W\right)}\right)$. The posterior distribution of $F_{j}^{*\left(M\right)}$ can be inferred using Equations 8, 9, 10, and 11 as follows:

(Equation 25) $$P\left(F_{j}^{*\left(M\right)}|Y^{\left(W\right)},Y^{\left(M\right)}\right)=\int P\left(F^{\left(M\right)},X^{\left(W\right)},U|Y^{\left(W\right)},Y^{\left(M\right)}\right)dX^{\left(W\right)}dU=\int \left[\int P\left(F^{\left(M\right)}|U,X^{\left(W\right)}\right)q\left(U\right)dU\right]q\left(X^{\left(W\right)}\right)dX^{\left(W\right)}=\int q\left(F^{\left(M\right)}|X^{\left(W\right)}\right)q\left(X^{\left(W\right)}\right)dX^{\left(W\right)}\text{.}$$

Although this integral is analytically intractable, Titsias and Lawrence and Damianou et al. showed that the mean and covariance of $F_{j}^{*\left(M\right)}$ can be calculated as follows:

and (Equation 26) $$E\left[F_{j}^{*\left(M\right)}\right]={\left(\Lambda^{T}\Psi_{1}^{\left(W\right)}\right)}_{j}$$

(Equation 27) $$Cov\left[F_{j}^{*\left(M\right)}\right]=\Lambda^{T}\left(\Psi_{2}^{\left(W\right)}-\Psi_{1}^{\left(W\right)}{\Psi_{1}^{\left(W\right)}}^{T}\right)\Lambda +\psi_{0}^{\left(W\right)}I-Tr\left(\left[{K_{uu}}^{-1}ー{\left(K_{uu}+{\beta \Psi }_{2}^{\left(M\right)}\right)}^{-1}\right]\Psi_{2}^{\left(W\right)}\right)I\text{,}$$

where

(Equation 28) $$\Lambda =\beta {\left(K_{uu}+\beta \Psi_{2}^{\left(M\right)}\right)}^{-1}{\Psi_{1}^{\left(M\right)}}^{T}Y^{\left(M\right)}\text{,}$$

(Equation 29) $$\Psi_{1}^{\left(W\right)}=<K_{Iu}{>}_{q\left(X^{\left(W\right)}\right)}\text{,}$$

(Equation 30) $$\Psi_{2}^{\left(W\right)}=<K_{Iu}{K_{Iu}}^{T}{>}_{q\left(X^{\left(W\right)}\right)}\text{,}$$

(Equation 31) $$\Psi_{1}^{\left(M\right)}=<K_{Ru}{>}_{q\left(X^{\left(M\right)}\right)},\text{and}$$

(Equation 32) $$\Psi_{2}^{\left(M\right)}=<K_{Ru}{K_{Ru}}^{T}{>}_{q\left(X^{\left(M\right)}\right)}\text{.}$$

#### ZENomix implementation

In our implementation, we selected the Matern 3/2 kernel for both kernel functions *k*(*x*,*y*) and *l*(*x*,*y*) with length scales of $1/\sigma_{k}^{f}\left(=\theta_{k}\right)$ and ${1/\sigma^{f}}_{l}\left(=\theta_{l}\right)$, respectively. To initialize the means of the posterior distributions *M*_*I*_ and *M*_*R*_, principal-component analysis (PCA) was used for the input data. Before the first step of ZENomix, all input data were *Z* scored, and $\sum_{h}F\left(q\left(X^{\left(h\right)}\right)\right)$ and *MMD*(*M*^(*W*)^,*M*^(*M*)^) were divided by their initial values for normalization. Table S2 shows the detailed initial parameter settings. For optimization, we used the L-BFGS-B algorithm implemented in SciPy and the Adam optimizer implemented in Optax. ZENomix supports GPU acceleration when Adam is selected as the optimization method.

### Data collection and pre-processing

#### Mouse OB data

Wild-type OB ST data were originally generated by Ståhl et al.^22^ Normalized data and a list of highly variable genes were downloaded from SpatialDB^55^ (http://www.spatialomics.org/SpatialDB/), and Rep11 was used for further analyses. The mouse AD OB ST dataset was downloaded from Mendeley Data^3^ (https://doi.org/10.17632/6s959w2zyr.1). Normalized mouse wild-type and AD data were used for further analyses (including pre-processing and integration with wild-type ST data). To integrate the wild-type ST data with the AD ST data, the spatial information of the AD ST data was ignored, and each spot was considered a simulated scRNA-seq data point. We selected highly variable genes from the wild-type reference downloaded from SpatialDB as landmark genes. Genes not included in the simulated scRNA-seq data or whose expression in the simulated scRNA-seq data was 0 were removed from the landmark genes measured in ISH data.

#### Zebrafish early embryo data

Zebrafish early embryo ISH data were downloaded from the Satija Lab homepage (https://satijalab.org/). For zebrafish early embryo scRNA-seq data, we downloaded the raw data from the Gene Expression Omnibus database (accession number GEO: GSE106587) and pre-processed them as previously described^34^ for both genotypes (wild type and *MZoep*). Genes not included in the scRNA-seq data or whose expression in the scRNA-seq data was 0 were removed from the landmark genes measured in ISH data. The geometric data were obtained from Cang et al.^56^

#### *D. melanogaster* embryo data

For the wild-type reference data, we used the modified FISH data generated by Sakaguchi et al.,^41^ originally obtained from BDTNP (D_mel_wt atlas_r2.vpc from http://bdtnp.lbl.gov) and DVEX (bdtnp.txt). For the *bcd*-KD reference data, we downloaded *bcd*-KD FISH data^40^ from Figshare (https://figshare.com/articles/dataset/A_gene_expression_atlas_of_a_bicoid_depleted_Drosophila_embryo/1270915) and used the cohort name 5:76–100 (the end of stage 5) as the reference. The FISH data were log scaled before the ZENomix procedure. Wild-type and *bcd*-KD scRNA-seq data were obtained from Sakaguchi et al.^41^ Both scRNA-seq datasets were pre-processed as previously described.^41^

#### Human PFC scRNA-seq and 10× Visium data (PFC_visium data)

Healthy control (HC) reference data were obtained from the 10× Visium ST dataset generated by Maynard et al.^31^ and downloaded from the Spatial LIBD website (https://research.libd.org/spatialLIBD/). AD reference data were obtained from Miyoshi et al.^32^ using sample V11Y24-118_C1. scRNA-seq data for both HC and AD conditions were obtained from Gabitto et al.^30^ Because the original dataset contained approximately 1.3 million cells, we subsampled 50,000 cells per condition while preserving the original cell-type composition. These subsampled datasets were used as inputs to ZENomix. All scRNA-seq and Visium datasets had been quality controlled and pre-processed in their respective original publications.

#### Human MTG scRNA-seq and MERFISH data (MTG_merfish data)

For both HC and AD conditions, MERFISH data generated by Gabitto et al.^30^ were used. The sample H21.33.011.Cx26.MTG.02.007.3.01.05 was used as the HC reference, and H21.33.015.Cx26.MTG.02.007.1.0 was used as the AD reference. scRNA-seq data for MTG were also obtained from Gabitto et al.^30^ As with PFC scRNA-seq data, we subsampled 50,000 cells per condition while preserving the original cell-type composition. MERFISH expression matrices were normalized using the *sc*.*pp*.*normalize_total* function with target_sum = 1e4, followed by log transformation via *sc*.*pp*.*log1p* function prior to the ZENomix procedure.

#### Human PFC scRNA-seq and 10× Xenium data (PFC_xenium data)

For both HC and AD conditions, 10× Xenium datasets of human frontal cortex tissue were downloaded from the 10× Genomics website (https://www.10xgenomics.com/datasets/xenium-human-brain-preview-data-1-standard). Following the Squidpy documentation (https://squidpy.readthedocs.io/en/stable/notebooks/tutorials/tutorial_xenium.html), we performed quality control (QC) using *sc*.*pp*.*filter_cells* with min_counts = 50 and *sc*.*pp*.*filter_genes* with min_cells = 5 for both conditions. Normalization was performed using the *sc*.*pp*.*normalize_total* with default settings, followed by log transformation via the *sc*.*pp*.*log1p* function prior to the ZENomix procedure. The scRNA-seq datasets used for integration with the Xenium data were identical to those used in the PFC_visium analysis.

### ZENomix and downstream analysis

ZENomix was built in Python 3.10.5 and is available on GitHub (https://github.com/yasokochi/ZENomix). All other software used in the ZENomix is publicly available: Numpy v.1.22.4 (https://numpy.org/) for calculation; scipy==1.9.3 (https://scipy.org/) for calculation; jax v.0.3.25 (https://jax.readthedocs.io/en/latest/index.html) for calculation; scikit-learn v.1.1.3 (https://scikit-learn.org/) for traditional machine learning (e.g., PCA); and pandas v.1.5.1 (https://pandas.pydata.org/) for reading data frames. For downstream analysis, we used Scanpy v.1.9.1^57^ (https://scanpy.readthedocs.io/en/stable/) for scRNA-seq data analysis and Squidpy v.1.2.3^58^ (https://squidpy.readthedocs.io/en/stable/) for spatial transcriptome data analysis.

### Calculating Moran’s I statistics

The accuracy of the predicted spatial transcriptomes was evaluated by comparing spatial autocorrelations (Moran’s I values) between the predicted and original spatial transcriptome data. Moran’s I value was calculated as follows:

(Equation 33) $$I=\frac{n}{W}\frac{\sum_{i=1}^{n}\sum_{j=1}^{n}w_{ij}\left(x_{i}-\bar{x}\right)\left(x_{j}-\bar{x}\right)}{\sum_{i=1}^{n}{\left(x_{i}-\bar{x}\right)}^{2}}\text{,}$$

where *n* is the number of data points indexed by *i* and *j*, *x* depicts the data point, $\bar{x}$ is the mean of *x*, *w*_*ij*_ is a matrix of spatial weights with zeroes on the diagonal, and *W* is the sum of all *w*_*ij*_. We used the *gr*.*spatial_autocorr* function with mode = “moran” in Squidpy for implementation.

### Calculating gene-wise Wasserstein distance

While Moran’s I captures similarities in spatial autocorrelation structures, it does not directly assess discrepancies in the overall distribution of gene expression levels. To complement this metric, we computed gene-wise Wasserstein distances between the predicted and measured spatial transcriptome datasets. The Wasserstein distance quantifies the minimal transport cost required to transform one gene expression distribution into another, thereby reflecting global distributional differences that may not be captured by autocorrelation measures. Wasserstein distances were calculated using the *wasserstein_distance* function implemented in SciPy.

### Sensitivity analysis of model parameters

To evaluate the influence of model parameters on the performance of ZENomix, we conducted a parameter importance analysis using Optuna,^59^ a Bayesian optimization framework that provides automated hyperparameter search and importance estimation. The number of optimization trials was set to 150 for the PFC_visium dataset and 100 for each of the MTG_merfish and PFC_xenium datasets.

### Robustness to sparsity of scRNA-seq data

The robustness of ZENomix to varying levels of scRNA-seq data sparsity was evaluated. To simulate different sparsity conditions, we introduced artificial dropout into the PFC scRNA-seq and mouse OB datasets. Specifically, gene expression values were randomly set to zero using predefined dropout rates ranging from 0.1 to 0.9. For each dropout level, the experiment was repeated with multiple random seeds to ensure robustness of the evaluation. The resulting PFC scRNA-seq data were used for HC prediction under a 10-fold holdout setting, whereas the resulting mouse OB simulated scRNA-seq data were used for zero-shot AD prediction.

### Analysis of the simulated mouse OB scRNA-seq data

Although the simulated scRNA-seq dataset was generated by masking spatial coordinates from the mouse OB ST data, each simulated data point may represent multiple cells, as individual ST spots capture transcripts from several cells. To assess whether this simulated dataset can reasonably serve as a substitute for true scRNA-seq data, we applied a standard scRNA-seq analysis pipeline using Scanpy. We first computed standard QC metrics, including total counts, the number of detected genes, and mitochondrial read fraction. After QC inspection, we applied Leiden clustering with a resolution parameter of 0.6. To evaluate the biological interpretability of the resulting clusters, we examined the expression patterns of known cell-type-specific marker genes and identified the top 10 DE genes for each cluster.

### Selecting spatially DE genes

We first calculated the maximum absolute value and standard deviation of expression differences between *MZoep*-mutant spatial transcriptomes and those of the wild type. Subsequently, 142 genes were selected by manually applying thresholds of 8 > *max_diff* > 2 and *SD* > 0.72. Based on the mean expression changes in the embryo margin, these genes were classified into two groups: putative NU and ND genes. The embryo margin was defined as tier = “1–2” or “3–4” in the zebrafish geometry data. The spatial gene expression profiles of the putative NU and ND genes were clustered via hierarchical clustering using the *cluster*.*hierarchy*.*fcluster* function in SciPy based on the correlation matrices of the gene expression changes, and four and three modules were obtained for the putative NU and ND genes, respectively. As module 1 of the putative NU genes and modules 1 and 3 of the putative ND genes showed a universal gene expression change, 87 spatially DE genes (74 and 13, respectively) were excluded.

### Statistical test of spatially DE genes

The statistical significance of the identified candidate ND genes was evaluated by comparing expression levels in the embryonic margin between wild-type and *MZoep* embryos. For each gene, a two-sided unpaired *t* test was performed, followed by BH correction for multiple testing. Genes were considered significant if they met both criteria: |log2 fold change| > 1.5 and an adjusted *p* < 0.05.

### Analysis using other methods (Perler, Tangram, and SpaGE)

We used Perler,^16^ Tangram,^17^ and SpaGE^15^ for comparison with ZENomix. We used the same number of metagenes as in the ZENomix experiments (*q* = 30 for mouse OB; *q* = 20 for zebrafish early embryo). All other parameter settings were left at their default values. For Tangram, we evaluated both the cells and clusters modes when cluster labels were available in the scRNA-seq data; otherwise, only the cells mode was used. Default parameter settings were applied across all datasets, including mouse OB, zebrafish early embryo, PFC_visium, MTG_merfish, and PFC_xenium. For SpaGE, we used the same number of principal vectors as in the ZENomix experiments whenever computationally feasible (*n*_pv = 30 for the mouse OB; *n*_pv = 20 for the zebrafish early embryo; *n*_pv = 50 for PFC_visium). Due to computational constraints, we set *n*_pv = 30 for both the MTG_merfish and PFC_xenium datasets.

### Data visualization

The publicly available tools Matplotlib v.3.6.2 (https://matplotlib.org/) and Seaborn v.0.12.1 (https://seaborn.pydata.org/) were used to visualize the data. For mouse OB data, we used the spatial location data from the collected wild-type and AD-mutant mouse OB ST data, as described above in the data collection and pre-processing section. To visualize the zebrafish embryo data, we used the plot_zf function created by Cang et al.,^56^ which interpolates the original 64 data points *in situ*. For the *Drosophila* embryo data, the embryos were visualized as previously described.^16^

## Resource availability

### Lead contact

For more information and resource requests, please contact Yasushi Okochi (okochi.yasushi.z8@f.mail.nagoya-u.ac.jp).

### Materials availability

This study did not generate new unique reagents.

### Data and code availability

This study is a reanalysis of existing data. The websites from which the data were collected are mentioned in the data collection and pre-processing subsection of the methods. ZENomix is developed under Python 3.10.5 and is available on GitHub (https://github.com/yasokochi/ZENomix) and Zenodo (DOI: https://doi.org/10.5281/zenodo.18656533).^60^

## Acknowledgments

We are grateful to Prof. Masahiko Hibi and Dr. Ken Nakae for their valuable discussions, Ms. Maiko Yokouchi for technical assistance, and Prof. Masahiko Hibi for kindly gifting pCS2-*lefty1*. This study was supported in part by the Moonshot R&D Program (JPMJMS2024-9 to H.N.) and CREST (JPMJCR25Q2 to H.N.) from the Japan Science and Technology Agency (JST), Japan Agency for Medical Research and Development (AMED) Multidisciplinary Frontier Brain and Neuroscience Discoveries (Brain/MINDS 2.0) (JP25wm0625322 and JP25wm0625210 to H.N.), and KAKENHI (21H03541 to H.N.; JP25K24423 to Y.O.) and Challenging Exploratory Research (grant numbers 22H02821 and 21K19265 to T.M.) from the JSPS.

## Author contributions

Y.O. and H.N. conceived the project. Y.O. developed the model. Y.O. and T.M. conducted the experiments. Y.O., S.S., and T.K. analyzed the data. Y.O. and H.N. wrote the manuscript with input from all the authors.

## Declaration of interests

The authors declare no competing interests.

## Declaration of generative AI and AI-assisted technologies in the writing process

During the preparation of this work, the authors used generative AI-powered tools in order to fix grammar and spelling mistakes. The authors have reviewed and edited the content as needed and take full responsibility for the content of the publication.

## Supplemental information

Document S1. Figures S1–S23 and Tables S1 and S2

Data S1. Convergence of MMD (fraction of final value to initial value) across multiple datasets

Data S2. *MZoep* ISH data used in Figures 3 and S15

Data S3. Pathway enrichment and GO analysis by Enrichr used in Figure S20

Data S4. Statistical test of our identified ND genes across multiple methods used in Figures S20 and S21

Document S2. Article plus supplemental information
