# Supplementary material for: Zero-shot reconstruction of mutant spatial transcriptomes
Source: Patterns (N Y). 2026 Mar 31;7(6):101521. doi: 10.1016/j.patter.2026.101521 (PMC13280724; doi:10.1016/j.patter.2026.101521)
Supplement: Document S1. Figures S1–S23 and Tables S1 and S2 [file mmc1.pdf]

Patterns, Volume *7*

Supplemental information

Zero-shot reconstruction of mutant spatial transcriptomes

Yasushi Okochi, Takaaki Matsui, Shunta Sakaguchi, Takefumi Kondo, and Honda Naoki

Supplemental Figures

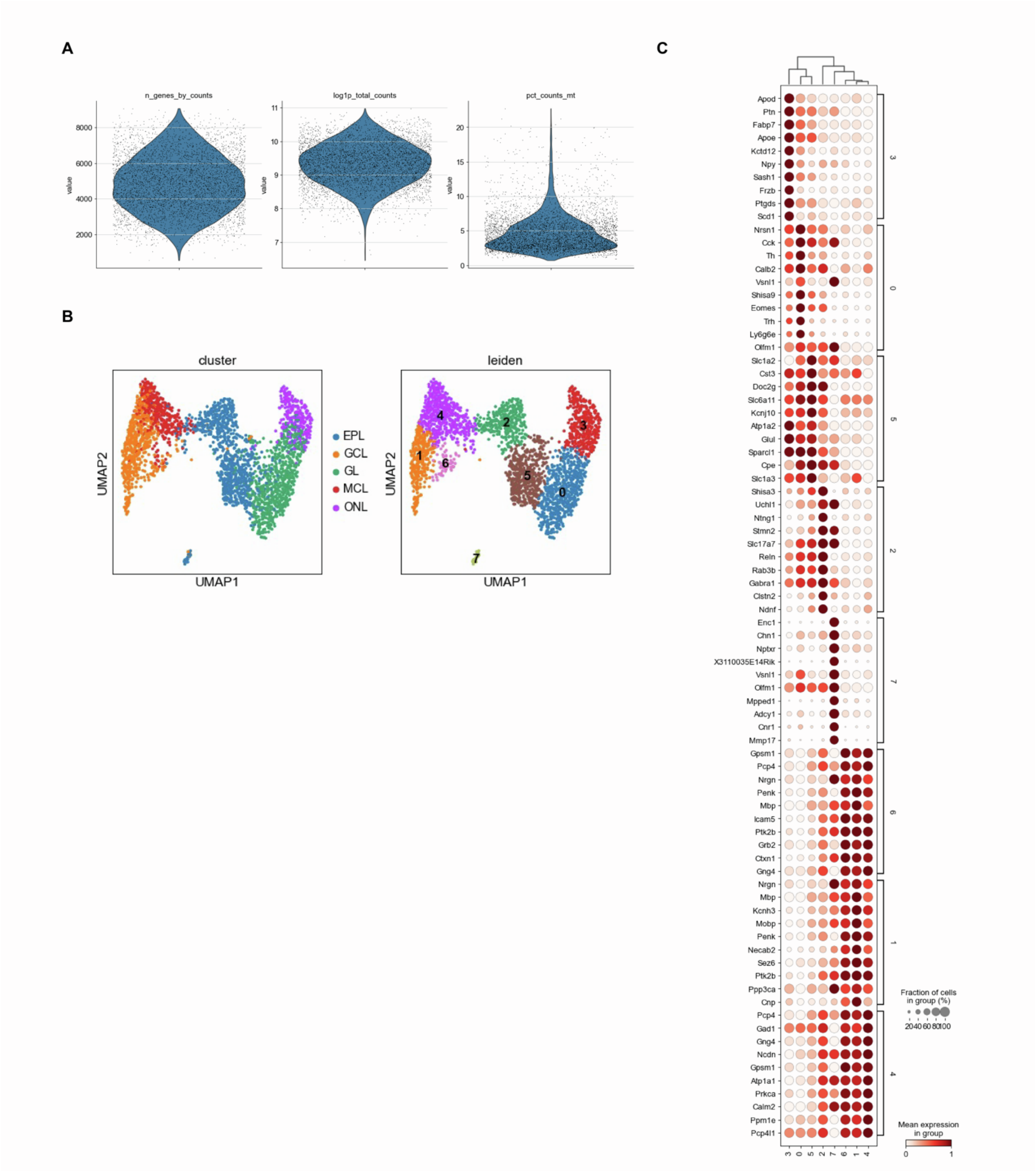

Figure S1. Quality control, clustering, and marker gene expression of simulated scRNA-seq data.

(**A**) Quality control metrics of the simulated single cells. Violin plots show the distribution of the number of genes detected per cell (left), total UMI counts per cell (center), and the percentage of mitochondrial transcripts (right). (**B**) Two-dimensional UMAP representation of the simulated scRNA-seq data. The UMAP is colored by the originally annotated cell classes (EPL, GCL, GL, MCL, ONL) and by Leiden clusters. (**C**) Dot plot of cluster-specific marker gene expression. Dot size represents the fraction of cells expressing each gene within a cluster, and dot color indicates the scaled mean expression level in the corresponding cluster. Genes are organized by hierarchical clustering, and clusters are shown along the horizontal axis. Cell types defined based on marker genes are as follows: Cluster 0 represents Periglomerular interneurons expressing

Cck and *Calb2*. Cluster 1 represents a mixed oligodendrocyte–neuron cluster expressing oligodendrocyte markers (*Mbp*, *Mobp*, *Cnp*) together with neuronal genes (*Nrgn*, *Penk*). Cluster 2 represents *Reln*/*Ndnf*-positive inhibitory interneurons characterized by *Reln* and *Ndnf*. Cluster 3 represents a mixed astrocyte–neuron cluster expressing astrocytic markers (*Apoe, Fabp7*) together with neuronal gene expression. Cluster 4 represents GABAergic interneurons expressing *Gad1*. Cluster 5 represents astrocytes expressing canonical astrocytic markers (*Slc1a2, Slc1a3, Glul, Kcnj10*). Cluster 6 represents a mixed oligodendrocyte–neuron cluster expressing *Mbp* together with neuronal markers (*Nrgn, Penk*). Cluster 7 represents excitatory projection neurons consistent with mitral/tufted cell populations expressing *Enc1, Nptxr*, and *Chn1*. Overall, five out of the eight clusters corresponded to a single cell type, including neurons or glia.

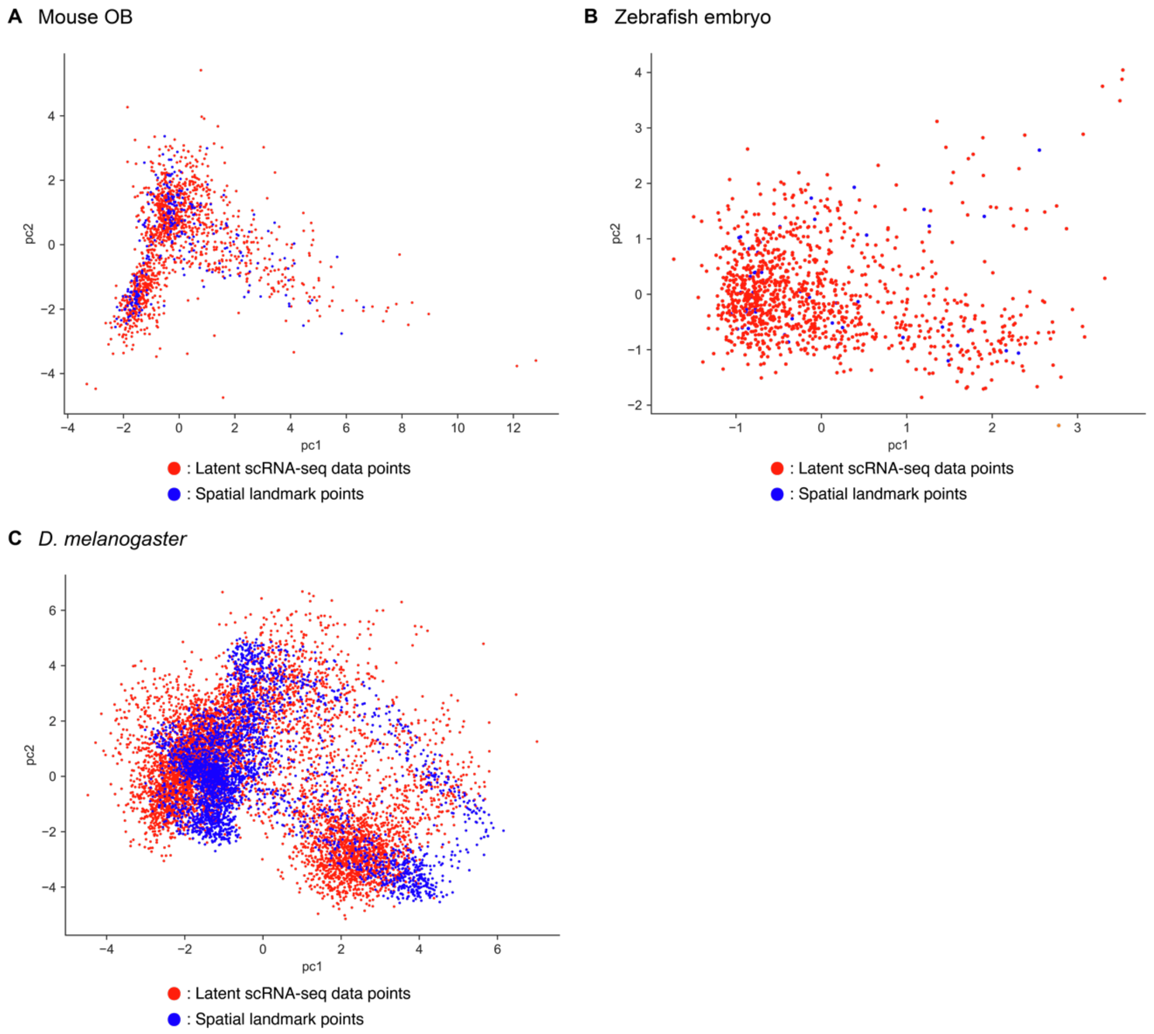

Figure S2. Latent data calibration for wild-type prediction

Scatter plots of calibrated distributions of spatial landmark and scRNA-seq data points (**Figure 2b**) for prediction of wild-type mouse OB (**A**), wild-type zebrafish embryo (**B**), and wild-type *D. melanogaster* embryo (**C**) spatial transcriptomes. Principal component analysis was used to visualise the latent space.

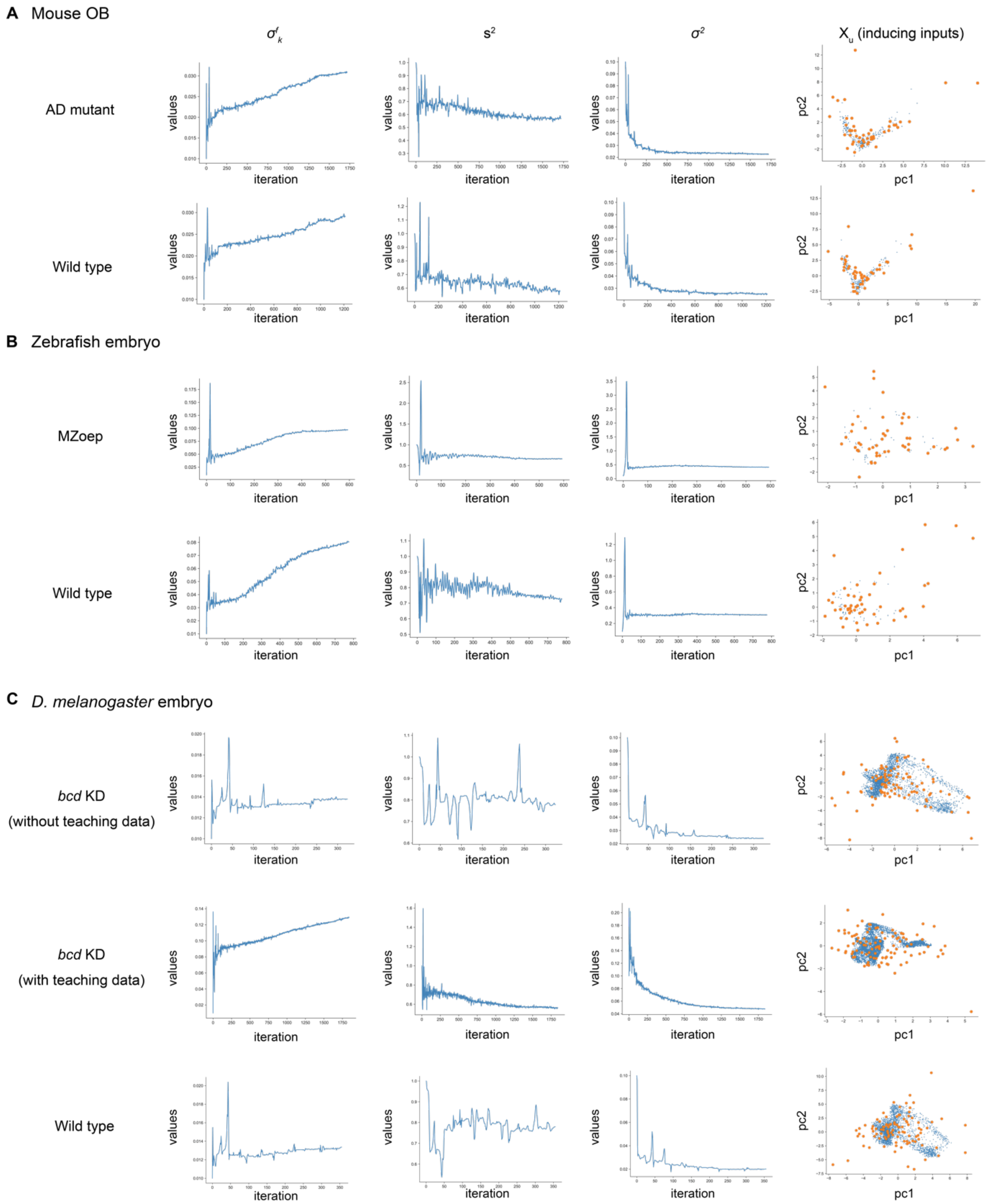

Figure S3. ZENomix parameter convergence

Parameter values during the first step of ZENomix for the prediction of mouse OB (**A**), zebrafish embryo (**B**), and *D. melanogaster* embryo (**C**) spatial transcriptomes. Each parameter is described in the **STAR Methods** section. Principal component analysis was used to show the final placements of the inducing points. The large orange and small blue points indicate the induced and spatial landmark points, respectively.

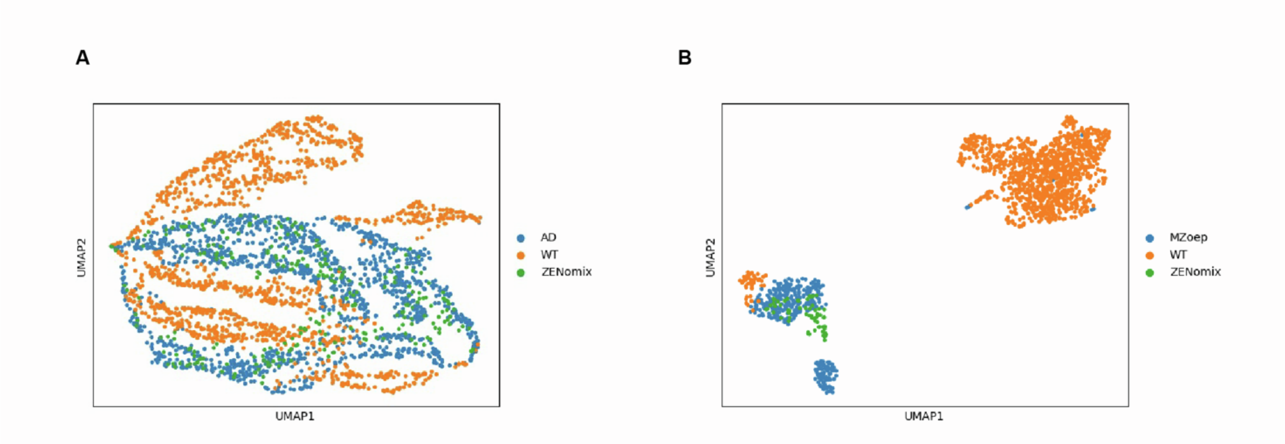

Figure S4. Projection of the reconstructed spatial transcriptomes by ZENomix to the UMAP

ZENomix's prediction was projected onto the UMAP embedding together with the original mutant and wildtype scRNA-seq data without any batch correction. (**A**) The predicted AD-mutant spatial transcriptomes, the original simulated AD-mutant and wildtype scRNA-seq data are shown. (**B**) The predicted *MZoep* spatial transcriptomes, the original *MZoep* and wildtype scRNA-seq data are shown.

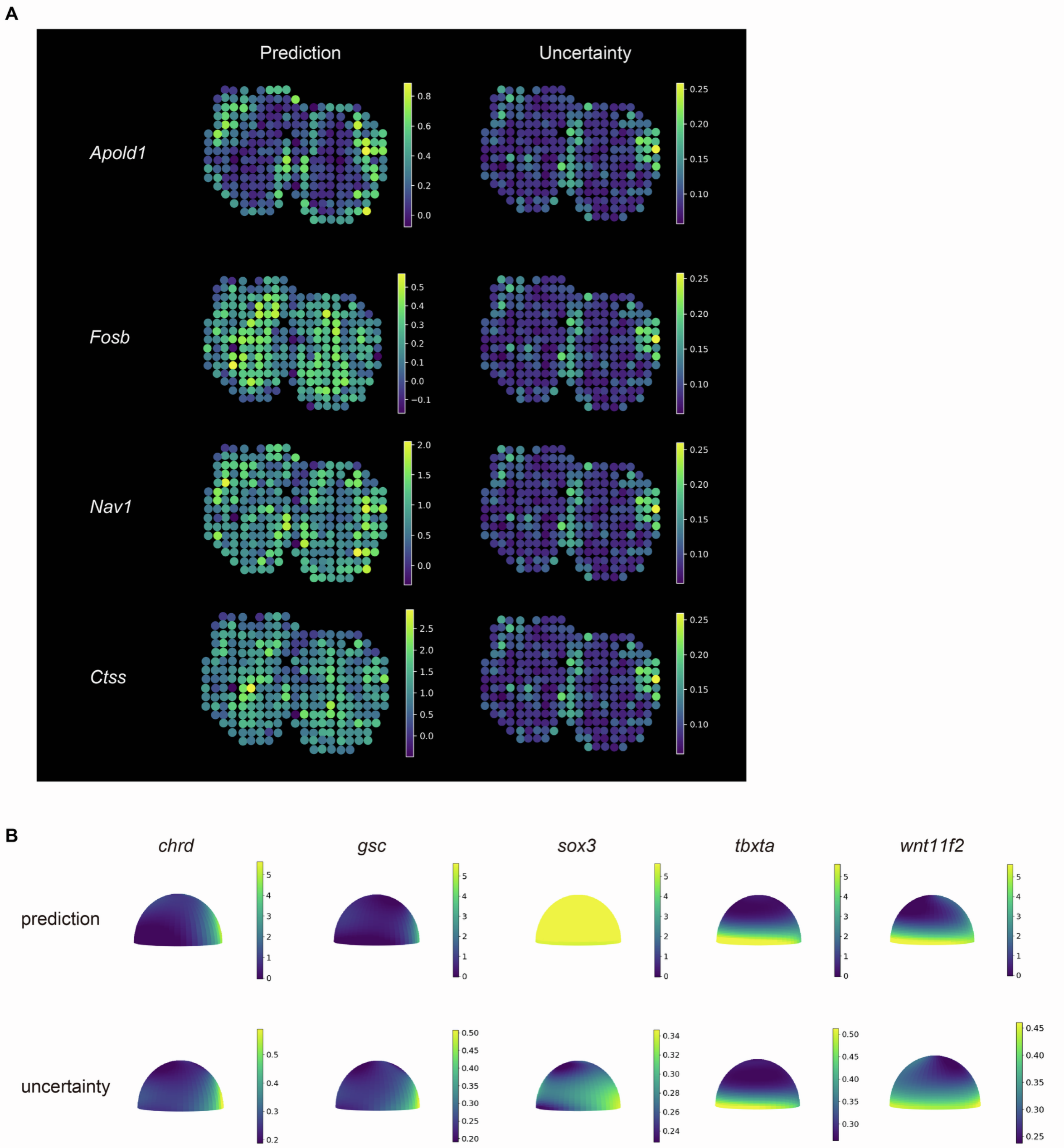

Figure S5. Predictive uncertainty of ZENomix

Predictive uncertainty of ZENomix of (**A**) AD-mutant mouseOB and (**B**) MZoep zebrafish early embryo was shown. Uncertainty was shown as standard deviation. Low uncertainty indicated that spatial transcriptomes were predicted with high confidence.

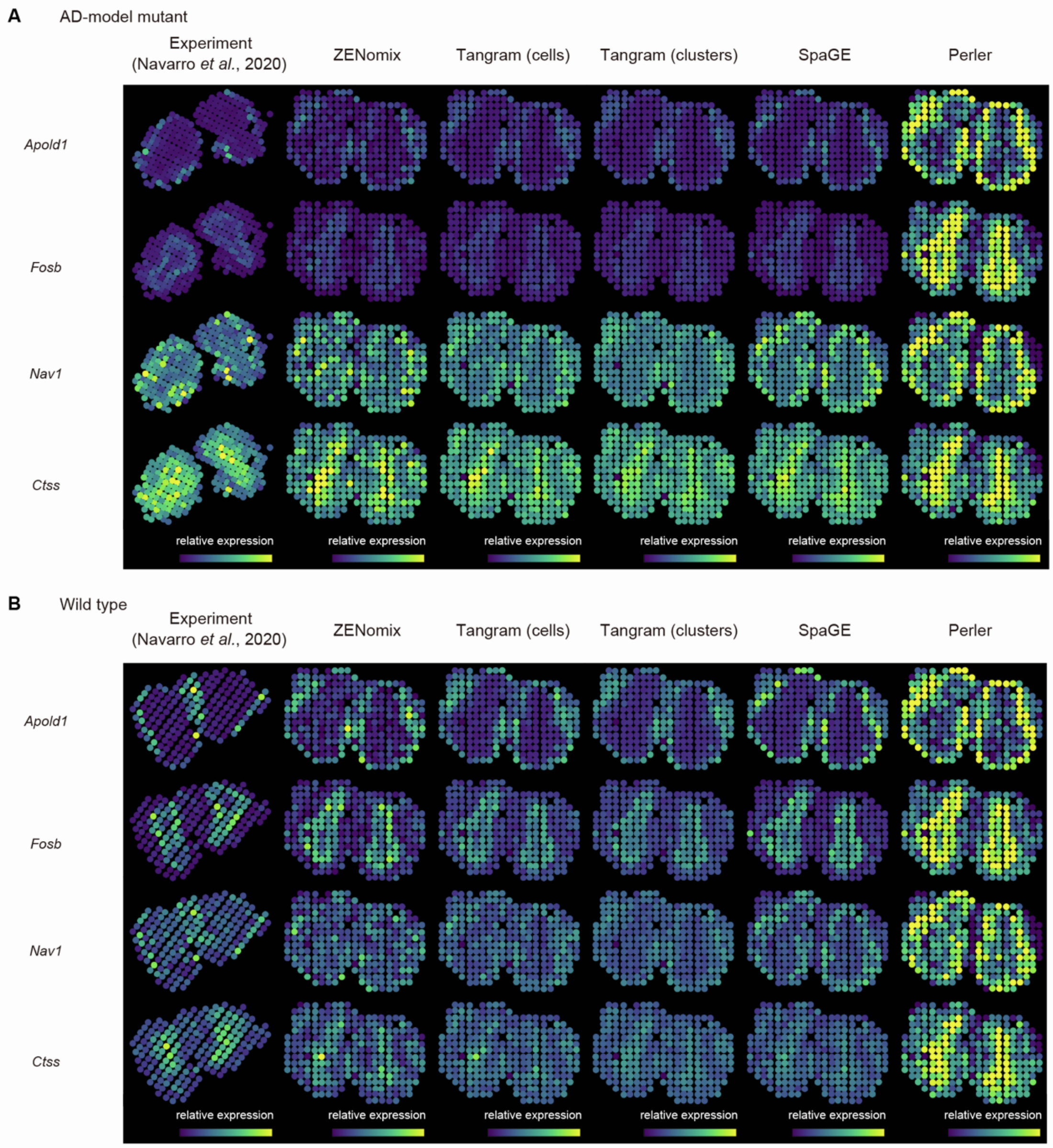

Figure S6. Prediction of mouse olfactory bulb data across ZENomix, Tangram, SpaGE, and Perler

Original and predicted spatial transcriptomes of (**A**) the AD-mutant and (**B**) wildtype mouse olfactory bulb across multiple methods.

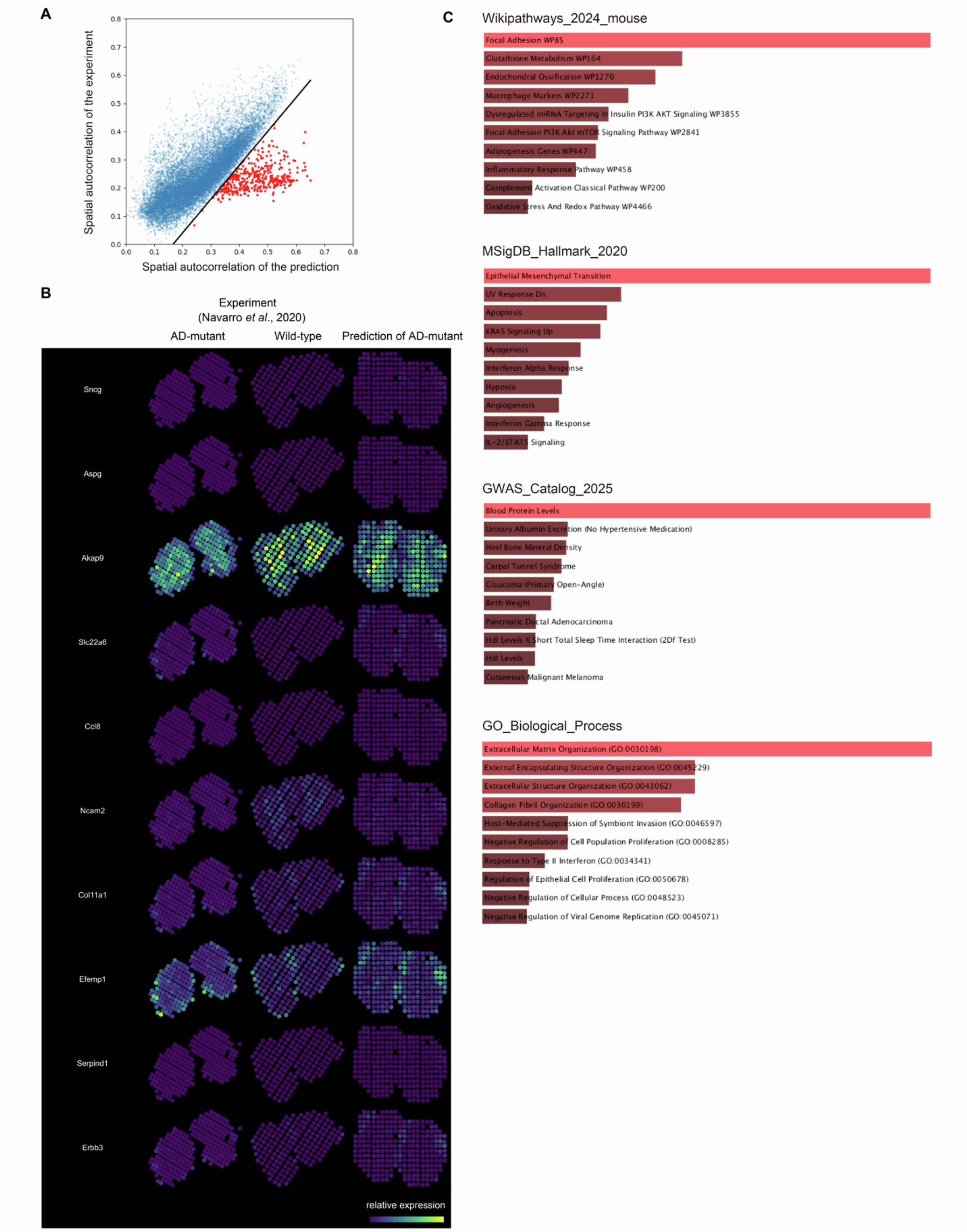

Figure S7. Poorly correlated genes in the mouse OB prediction

(**A**) Poorly correlated genes were manually selected. Red points indicate the selected poorly correlated genes. (**B**) Comparison of spatial gene expressions of ten randomly selected poorly correlated genes. The color bar is shared for comparison. (**C**) Enrichment analysis of the poorly correlated genes. Notably, no known AD-related biological processes were enriched among these genes. Panels correspond to the following annotation databases: Wikipathways (2024, mouse), MSigDB Hallmark (2020), GWAS Catalog (2025), and GO: Biological Process.

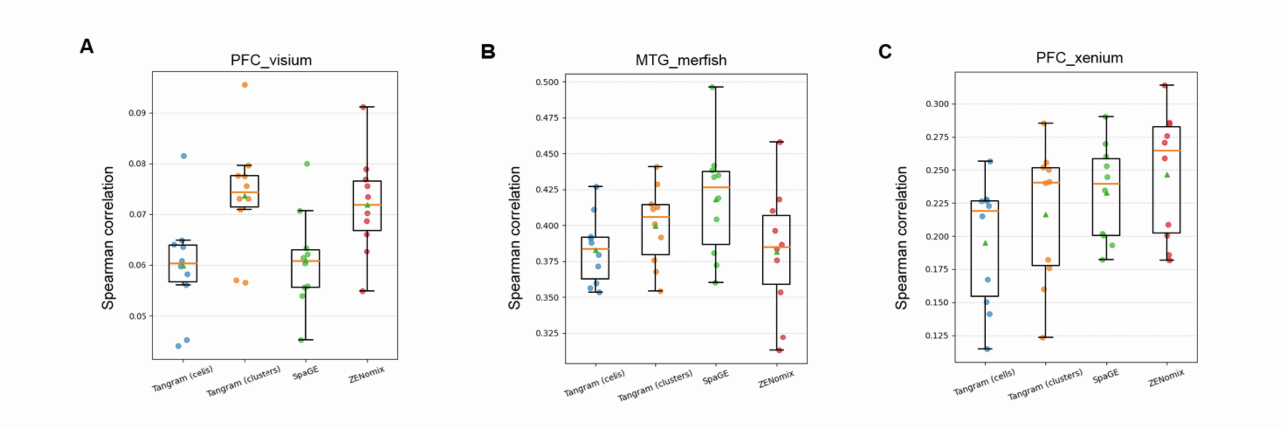

Figure S8. Performance comparison across three human brain datasets under the same genotype setting

(A–C) Performance comparison of wildtype prediction under the same genotype setting across ZENomix, Tangram in cells mode (Tangram (cells)), Tangram in clusters mode (Tangram (clusters)), and SpaGE. Performance was evaluated using 10-fold holdout experiments based on Spearman's correlation. Panels correspond to the following datasets: PFC_visium (**A**), MTG_merfish (**B**), and PFC_xenium (**C**). In PFC_visium dataset, ZENomix outperformed both Tangram (cells) and SpaGE. While Tangram (clusters) showed comparable performance to ZENomix, it relies on cluster-averaged expression and therefore ignores variability at the single-cell level. In the MTG_merfish dataset, SpaGE achieved the highest performance, and ZENomix and Tangram showed similar performance. In PFC_xenium dataset, ZENomix outperformed the other methods.

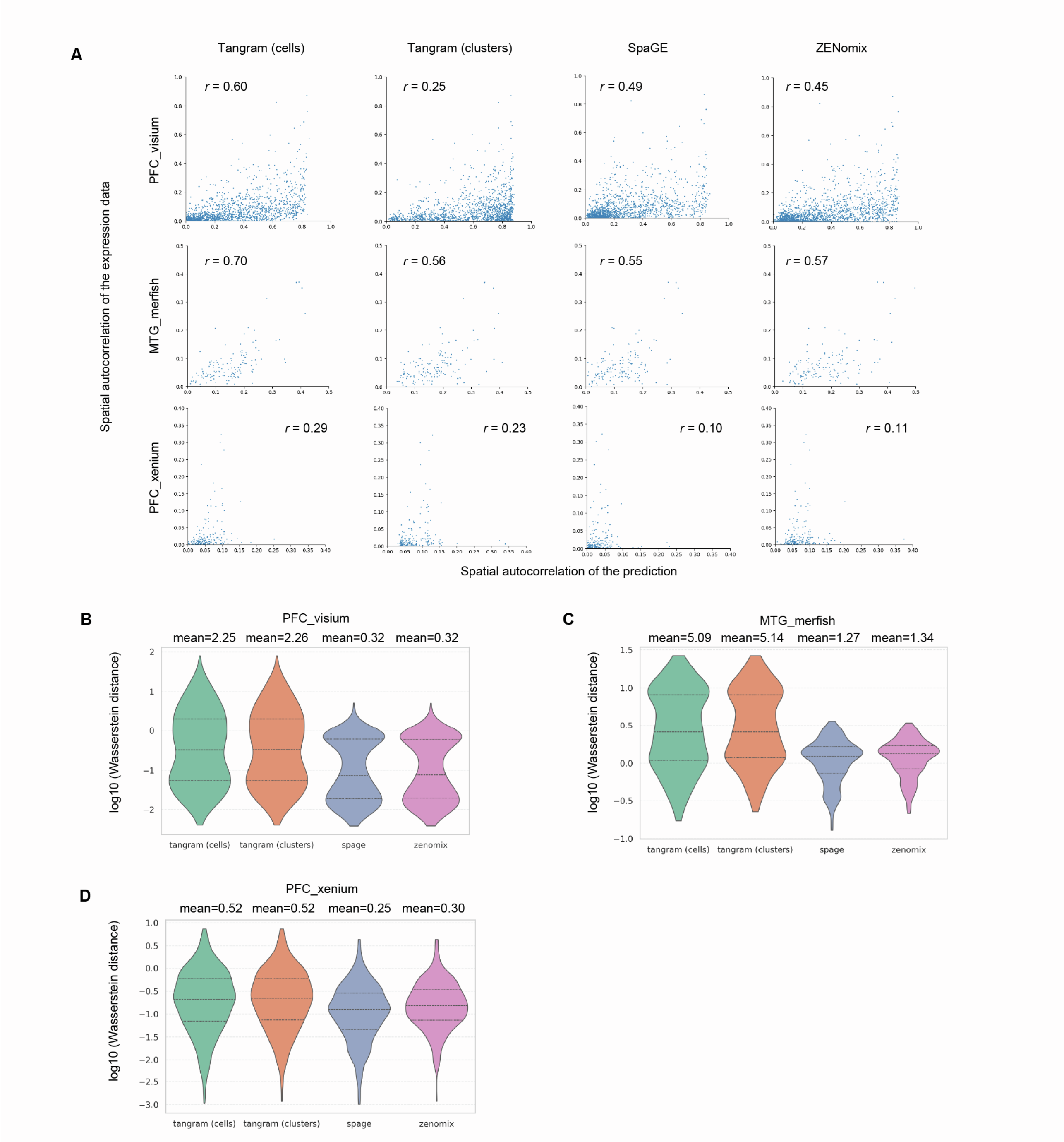

Figure S9. Performance comparison across three human brain datasets under the cross-genotype setting

(**A**) Performance comparison of AD prediction across ZENomix, Tangram, and SpaGE using Moran's I. (**B–D**) Performance comparison of AD prediction across ZENomix, Tangram, and SpaGE using gene-wise Wasserstein distance. Panels correspond to datasets as follows: PFC_visium (**B**), MTG_merfish (**C**), and PFC_xenium (**D**). Across all datasets, ZENomix and SpaGE showed comparable performance among these two metrics across datasets, whereas Tangram in both cells and clusters mode exhibited inconsistent behavior across these metrics.

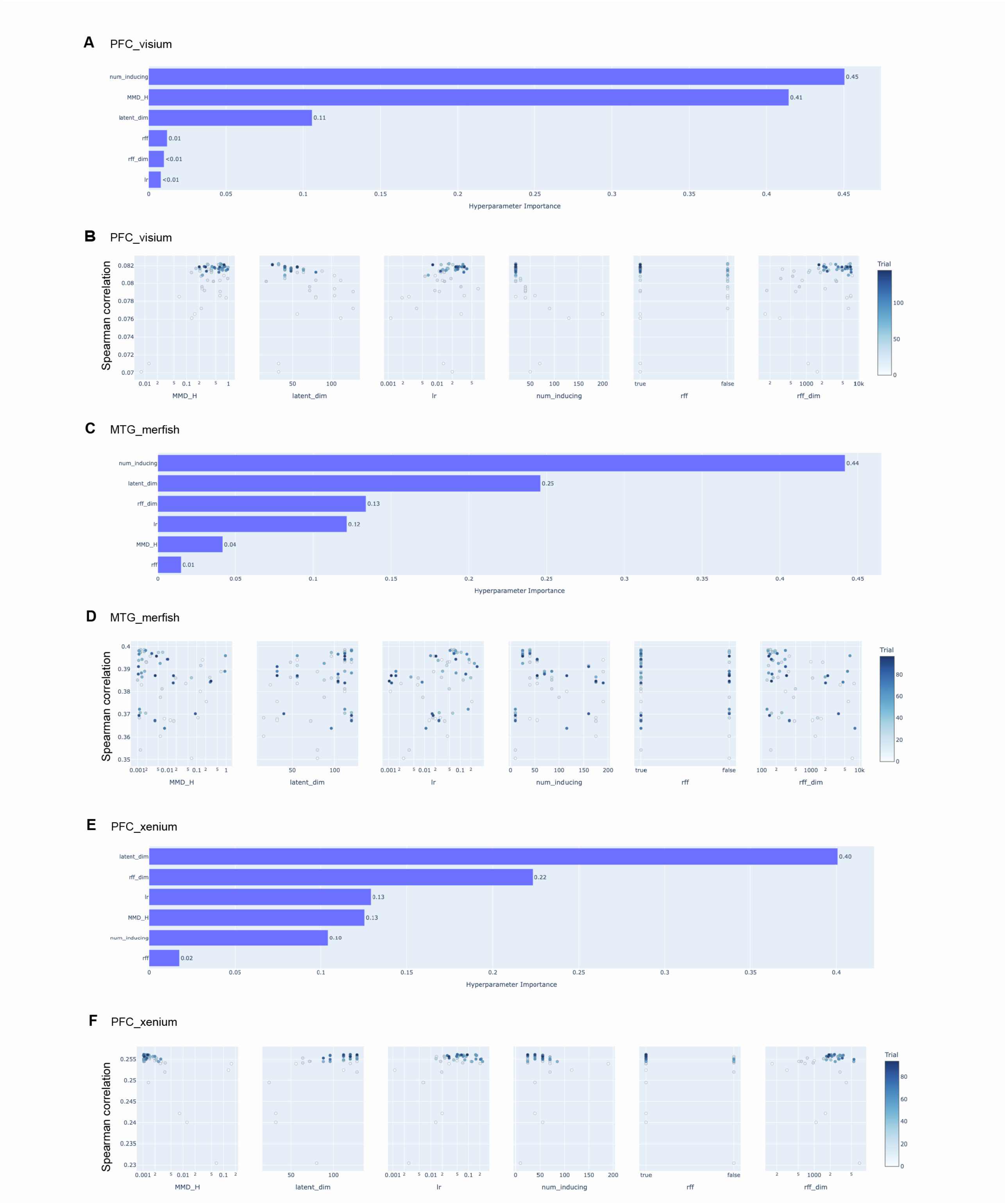

Figure S10. Sensitivity analysis of ZENomix across datasets

(**A**–**F**) Hyperparameter sensitivity analysis for PFC_visium, MTG_merfish, and PFC_xenium datasets using wild-type prediction. (**A**, **C**, **E**) Bar plots show the relative importance of each hyperparameter across Optuna optimization trials. Higher values indicate greater contribution to model performance. Each parameter name of ZENomix package corresponds to parameter described in STARMethods as follows: “num_inducing” is the number of inducing points, “latent_dim” is number of latent dimensions, and “MMD_H” is MMD kernel hyperparameter. “rff” is Boolean parameter, which controls the use of RFF approximation and “rff_dim” is number of RFF dimension. (**B**, **D**, **F**) Scatter plots depict the

relationship between individual hyperparameter values and model performance. Each point represents an Optuna trial, with color indicating trial index. Panels correspond to datasets as follows: PFC_visium (**A**–**B**), MTG_merfish (**C**–**D**), and PFC_xenium (**E**–**F**). These results indicate that the number of inducing points and the latent space dimensionality are important for the performance of ZENomix.

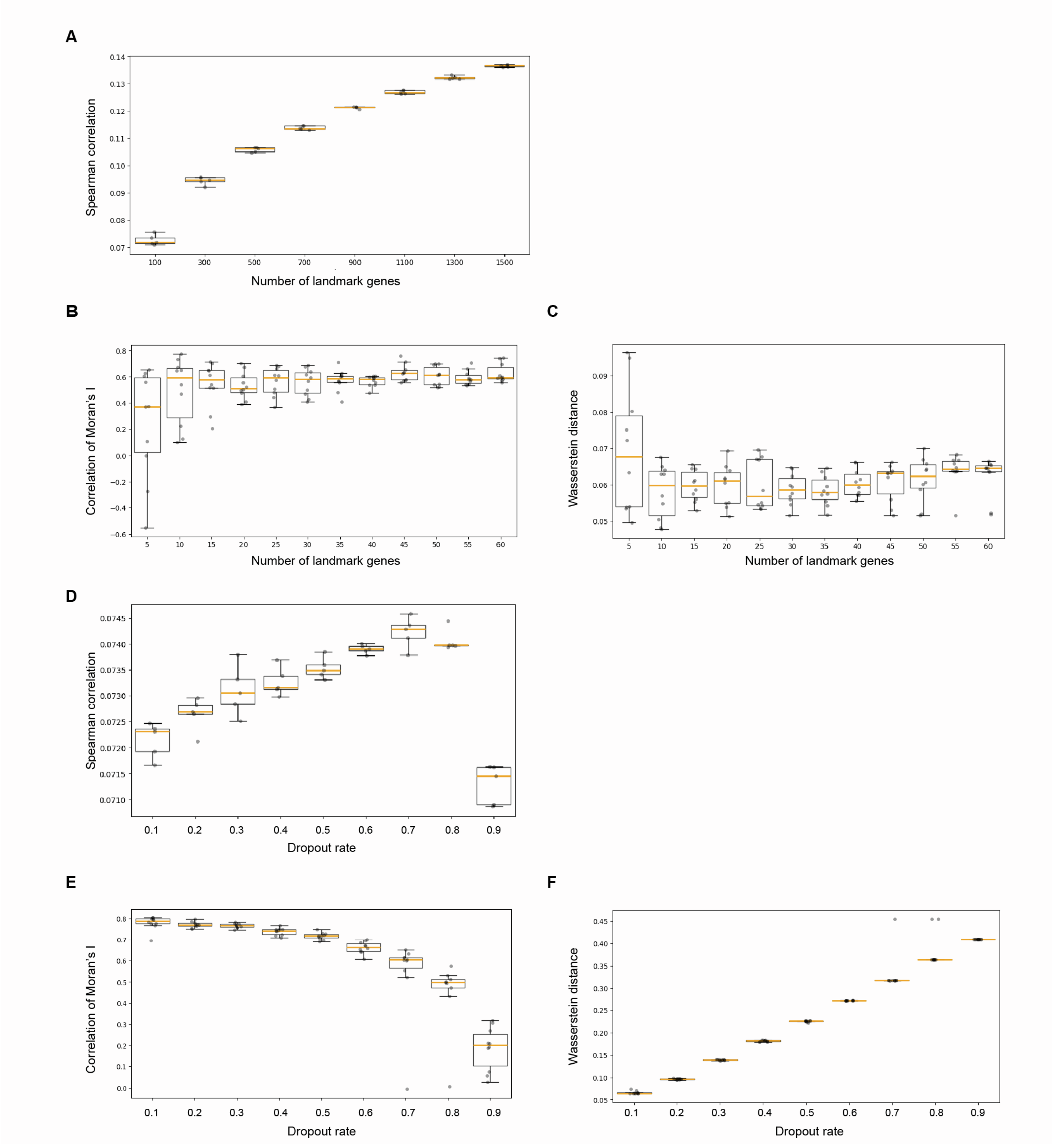

Figure S11. Effect of landmark gene number and scRNA-seq sparsity on ZENomix performance

Systematic evaluation of ZENomix performance under varying numbers of landmark genes (**A–C**) and scRNA-seq sparsity (**D–F**). Each dot indicates a random sampling seed. (**A**) Performance of ZENomix by randomly down-sampling different numbers of landmark genes for the PFC_visium datasets in the same-genotype settings. The performance remained stable up to around 900 landmark genes. (**B–C**) mouse OB AD-mutant prediction performance of ZENomix by randomly down-sampling different numbers of landmark genes in the cross-genotype settings. Performance was evaluated by spatial autocorrelation (**B**) and Wasserstein distance (**C**). The performance remained stable up to around 30 landmark genes. (**D**) Performance of ZENomix by introducing controlled dropouts to the scRNA-seq data for the PFC_visium datasets in the same-genotype settings. Although correlation slightly increased with increasing dropout rate, the reason for which remained unclear, the overall performance change was small, indicating the robustness of ZENomix. (**E–F**) Mouse OB AD-mutant prediction performance of ZENomix by introducing controlled dropouts to the simulated scRNA-seq data in the cross-genotype settings. Performance was evaluated by spatial autocorrelation (**E**) and Wasserstein distance (**F**). As

expected, the performance of ZENomix decreased as increasing dropout rate and spatial autocorrelation remained stable up to 0.5 of dropout rate.

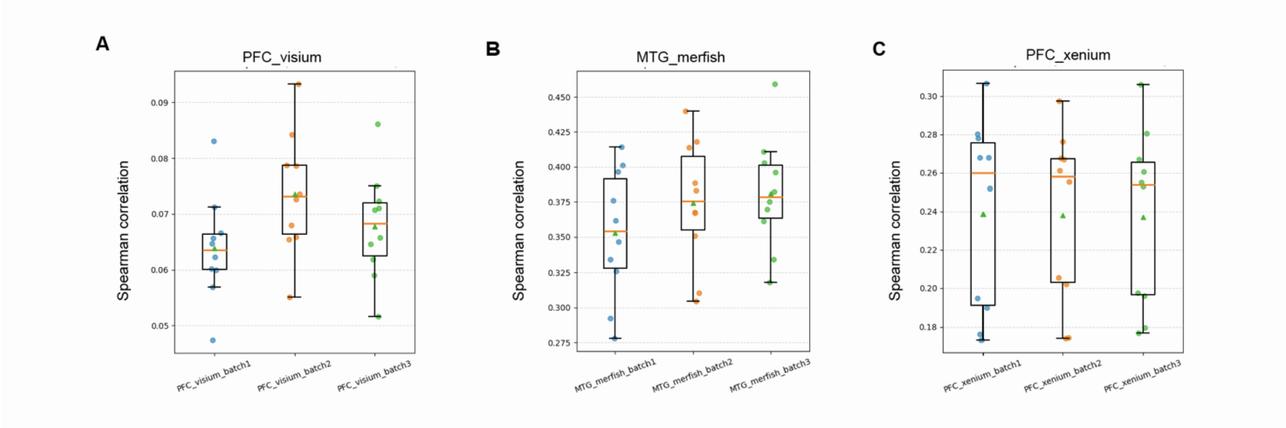

Figure S12. Robustness across scRNA-seq batches and ST platform shifts under the same genotype setting

(A–C) Performance comparison of wild-type prediction by ZENomix under the same genotype setting across three scRNA-seq batches for each dataset. Performance was evaluated using 10-fold holdout experiments based on Spearman's correlation. Panels correspond to the following datasets: PFC_visium (**A**), MTG_merfish (**B**), and PFC_xenium (**C**). In all datasets, ZENomix exhibited robust performance across different scRNA-seq batches.

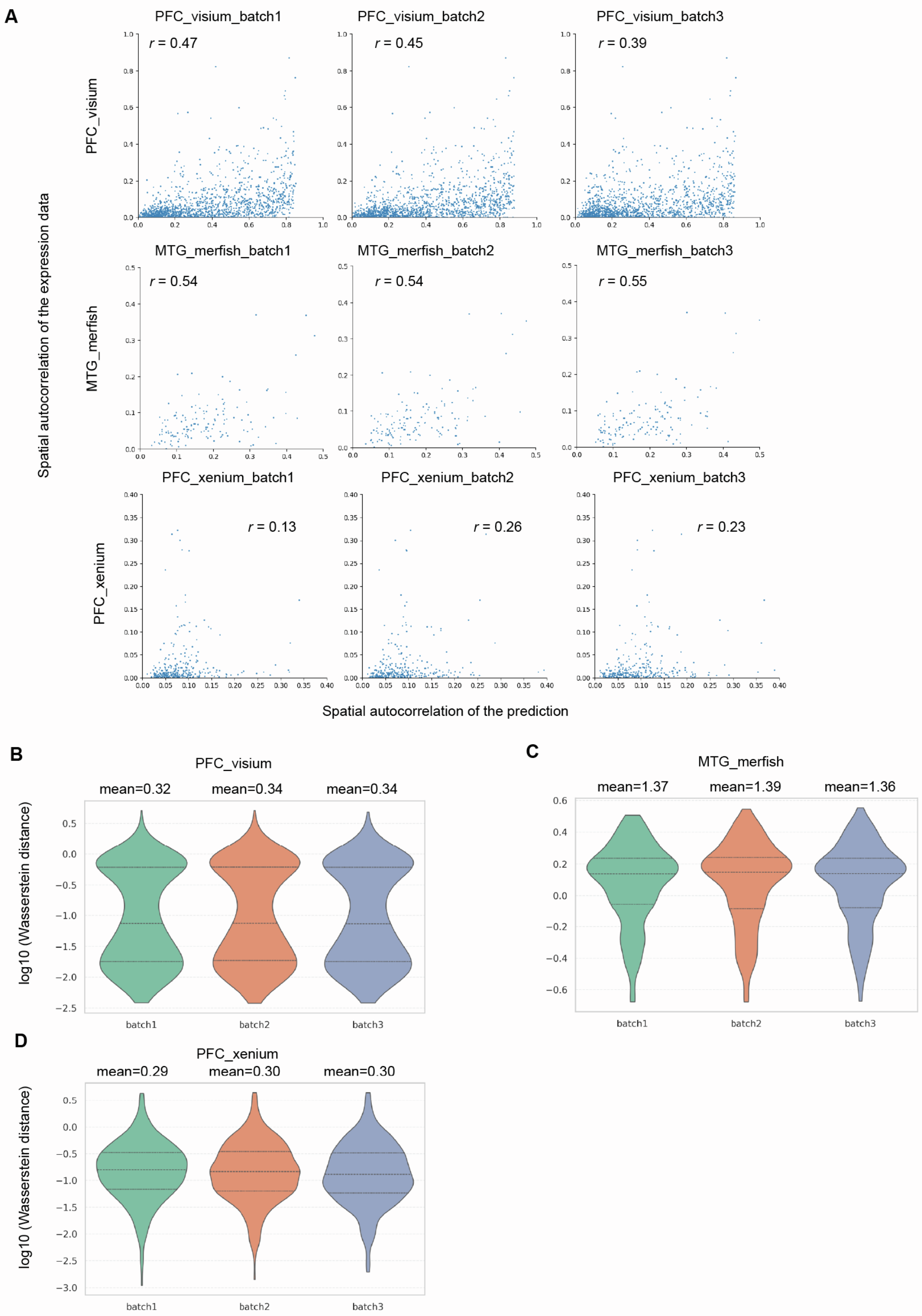

Figure S13. Robustness across scRNA-seq batches and ST platform shifts under the cross-genotype setting

(**A**) Performance comparison of AD prediction by ZENomix across three scRNA-seq batches for each dataset using Moran's I. (**B–D**) Performance comparison of AD prediction across three scRNA-seq batches for each dataset using gene-wise Wasserstein distance. Panels correspond to the following datasets: PFC_visium (**B**), MTG_merfish (**C**), and PFC_xenium (**D**). In all datasets, ZENomix exhibited robust performance across different scRNA-seq batches.

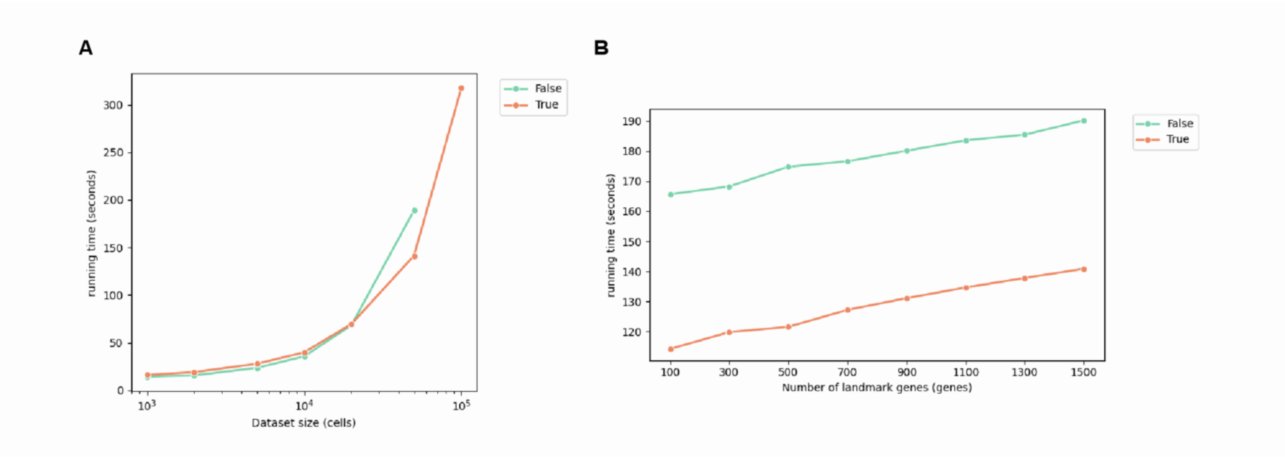

Figure S14. Running times for ZENomix

Running times of ZENomix procedures, including posterior inference and spatial reconstruction, were evaluated with respect to scRNA-seq data size (**A**) and number of landmark genes (**B**). The x-axis in **A** is shown on a logarithmic scale. The green line indicates ZENomix without RFF approximation, whereas the orange line indicates ZENomix with RFF approximation. The PFC_visium dataset was used. Experiments were conducted on a machine equipped with AMD Ryzen Threadripper PRO 3955WX (16 cores, 128 GB RAM) CPU and one NVIDIA RTX A6000 GPU with 48 GB VRAM. ZENomix without RFF approximation ran out of memory (48 GB VRAM) at 100k cells and ZENomix with RFF approximation ran out of memory at 200k cells. The RFF dimension was set to 2048.

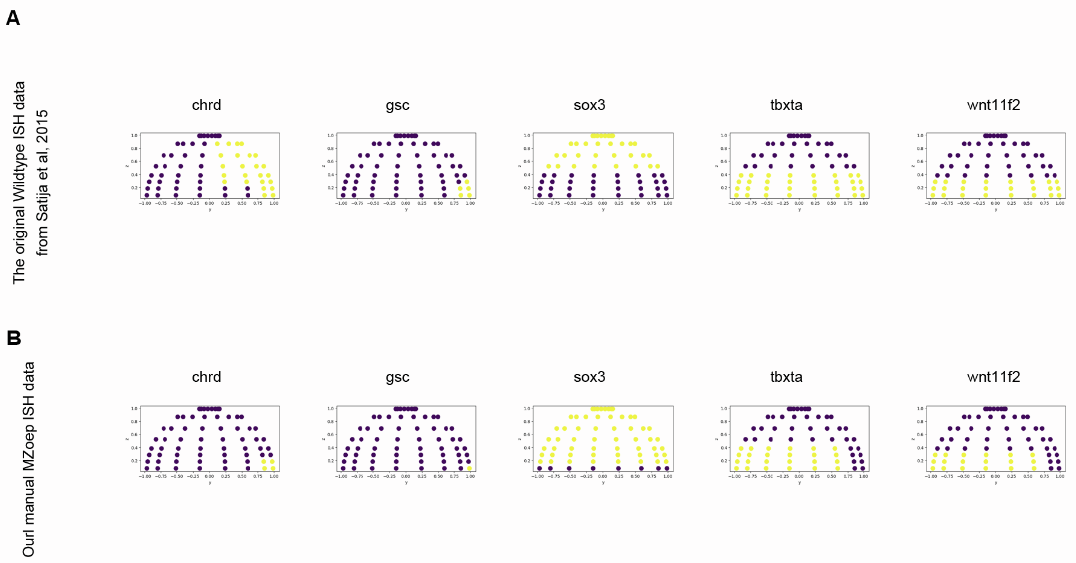

Figure S15. *MZoep* ISH data

(**A**) Ground-truth wild-type ISH data from Satija et al. used in Figure 3. (**B**) Ground-truth *MZoep* ISH data for *chrd, gsc, sox3, tbxta,* and *wnt11f2* used in Figure 3. ISH data were manually generated by binarizing the original ISH image from Grisman et al. and Bennett et al. Yellow dots indicate expression of the gene of interest.

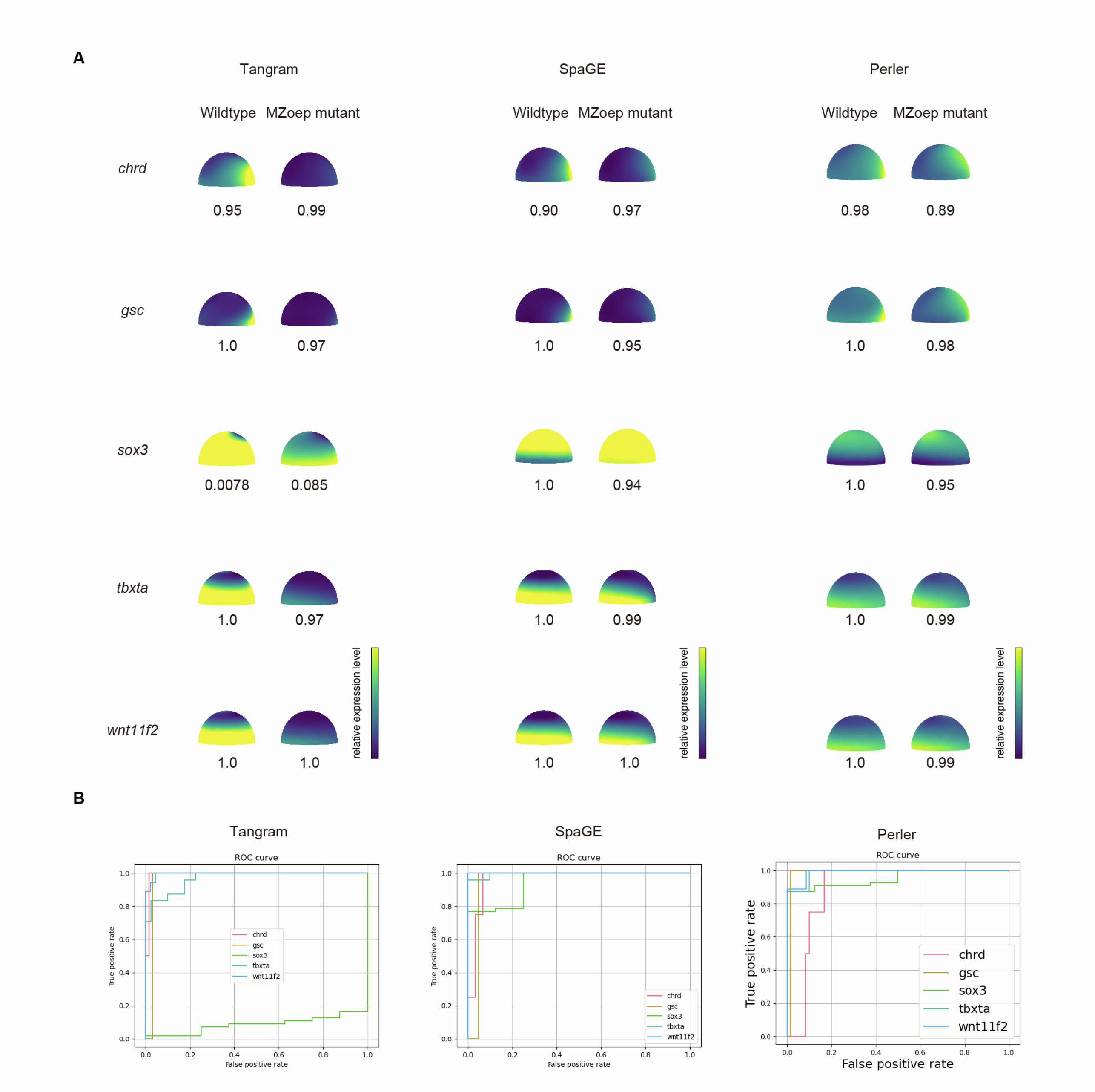

Figure S16. Prediction of zebrafish early embryo spatial transcriptomes using Tangram, SpaGE, and Perler

(**A**) Predicted spatial transcriptomes of *MZoep*-mutant and wild-type embryos. Corresponding to Figure 3, ROC scores are reported. The color bar is shared within each method. Notably, although Perler achieved a high ROC score, the prediction scales differed between MZoep and wild-type embryos. Tangram's prediction of *sox3* was flipped. SpaGE produced predictions largely consistent with those of ZENomix. (**B**) ROC curves for *MZoep*-mutant prediction of the genes shown in (**A**) across multiple methods.

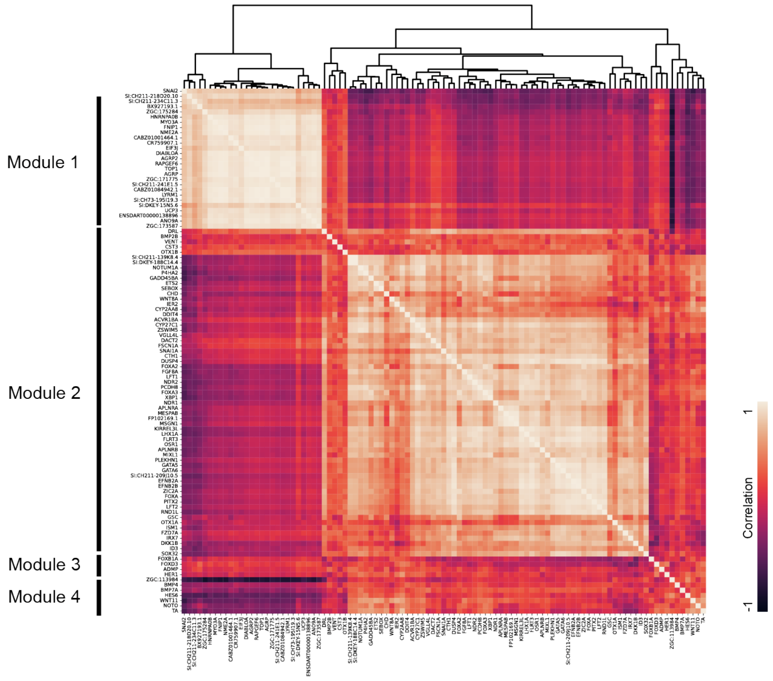

Figure S17. Putative NU gene hierarchical clustering

Hierarchical clustering of the putative NU genes (corresponding to **Figure 5d**). The heatmap indicates the correlations among the changes in the expression of newly screened NU genes.

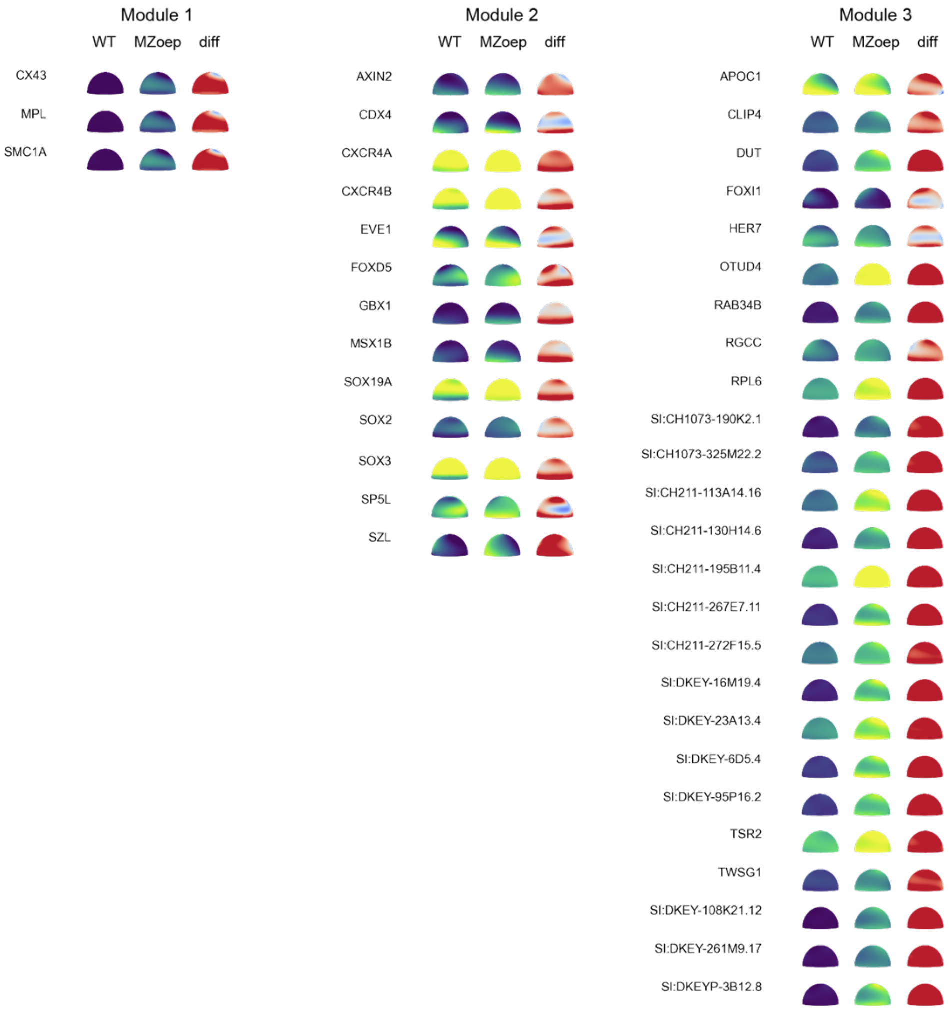

Figure S18. All putative NU genes

The predicted spatial gene expression patterns of wild-type and MZoep zebrafish embryos for all putative NU genes. ‘diff’ indicates the expression difference between the MZoep and wild-type spatial transcriptomes.

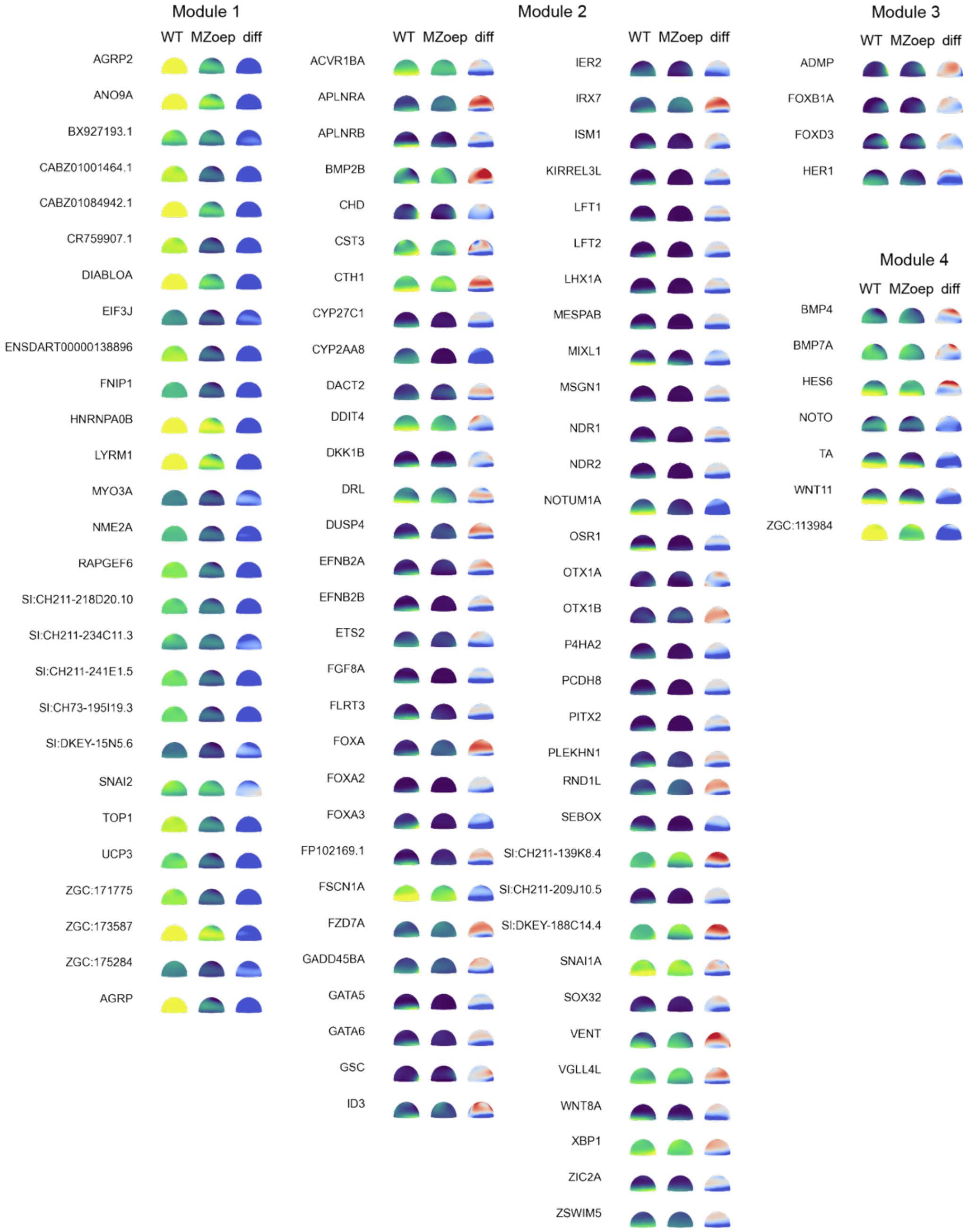

Figure S19. All putative ND genes

Predicted spatial gene expression patterns in wild-type and MZoep zebrafish embryos for all putative ND genes. ‘diff’ indicates the expression difference between the MZoep and wild-type spatial transcriptomes.

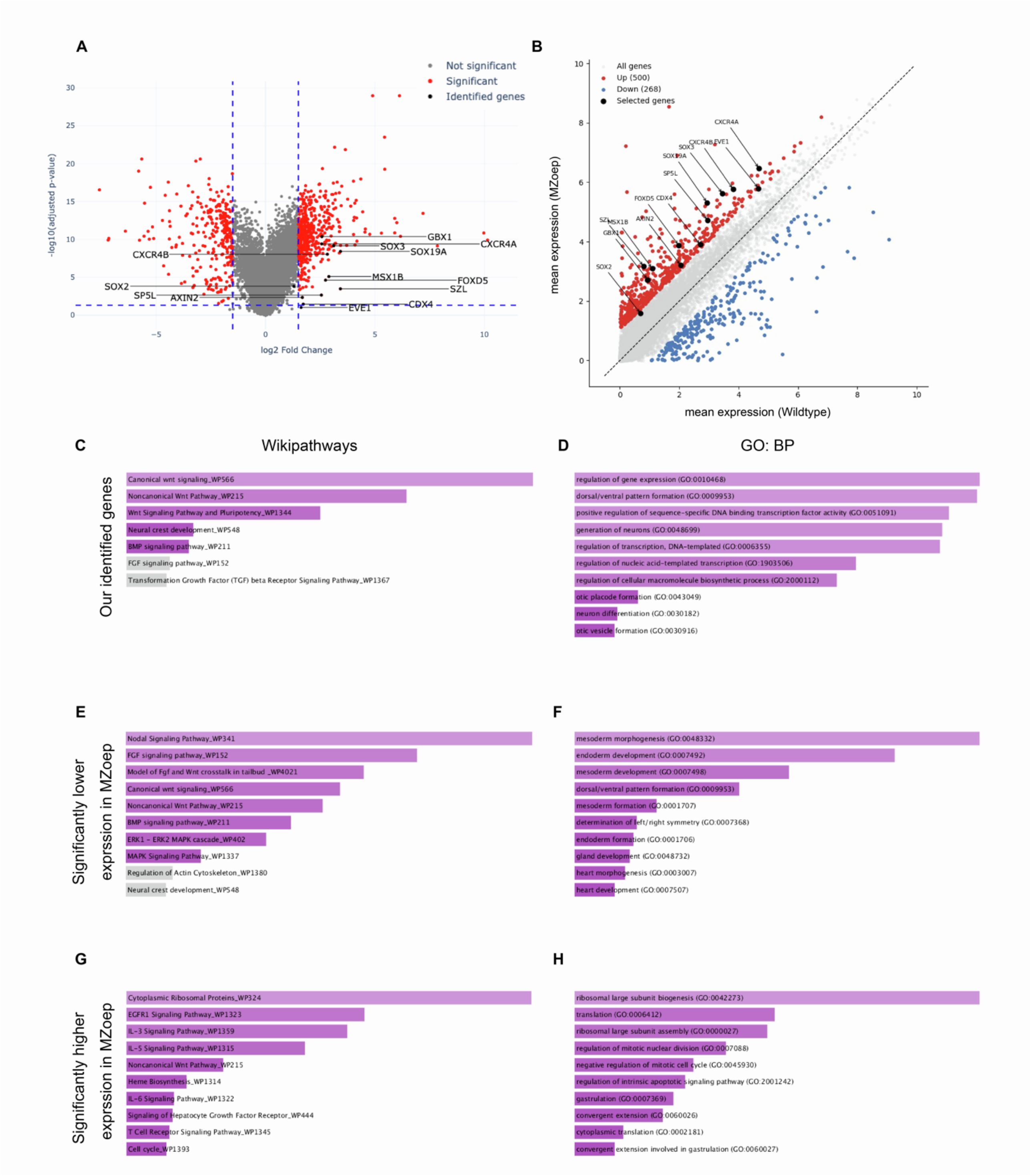

Figure S20. Statistical analysis of spatially DE genes

(**A**) Volcano plots showing spatially differential gene expression between wildtype and *MZoep* embryos inferred by ZENomix. (**B**) Scatter plots comparing mean expression levels between wildtype and *MZoep* embryos by ZENomix. Genes with higher expression in *MZoep* embryos (SHE genes) are shown in red, genes with lower expression genes (SLE genes) in blue, and our identified candidate ND genes are annotated. The diagonal line represents equal expression between conditions. Plot includes the total number of upregulated (SHE) and downregulated (SLE) genes identified. (**C–H**) Pathway enrichment analysis by FishEnrichr for our identified ND genes (**C, D**), the higher expressed genes (**E, F**), and the lower expressed genes (**G, H**). Panels correspond to the following annotation databases: Wikipathways (**C**, **E, G**), GO: Biological Process (**D, F, H**).

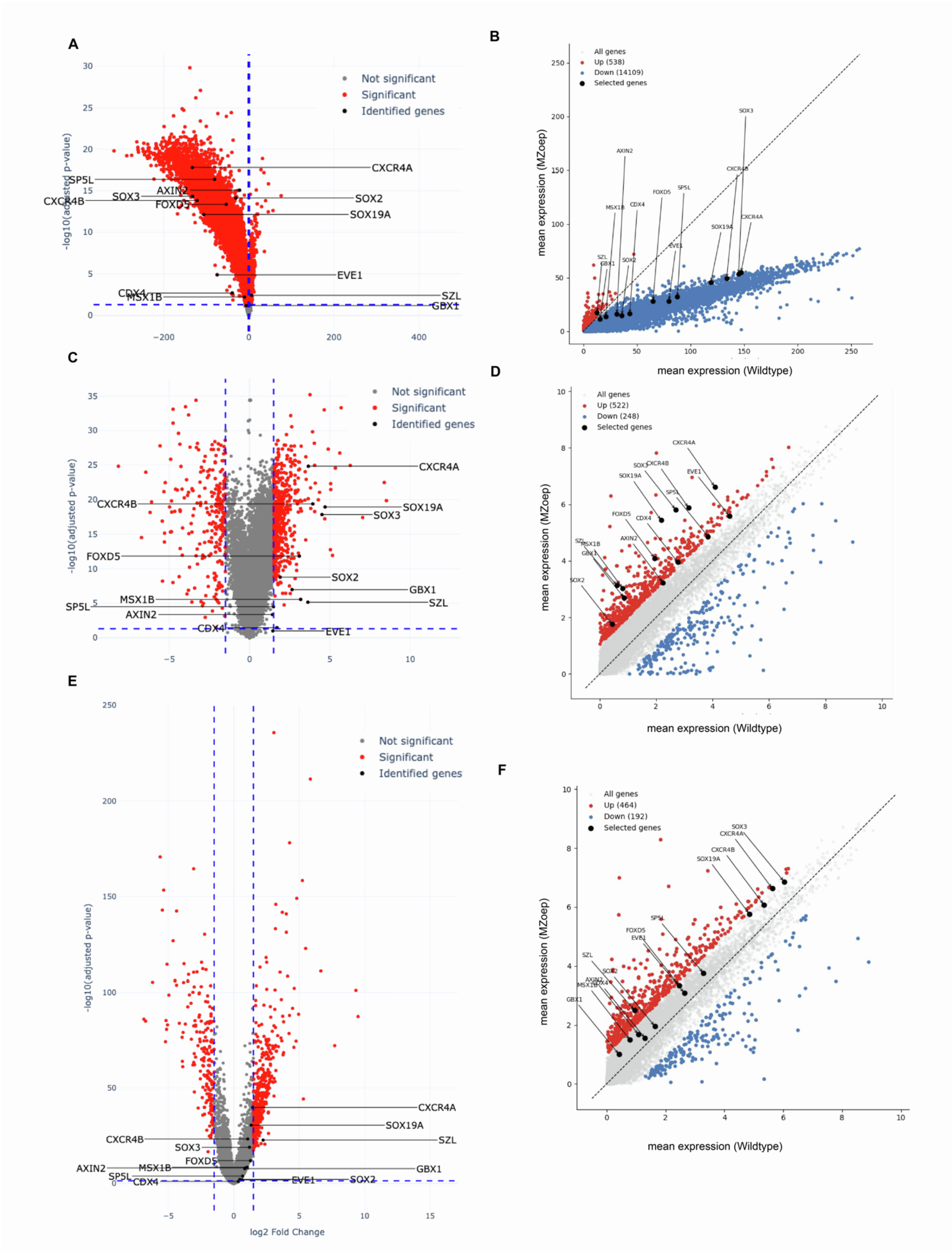

Figure S21. Comparison of spatially DE analysis

(**A, C, E**) Volcano plots showing spatially differential gene expression between wildtype and *MZoep* embryos inferred by Tangram (**A**), SpaGE (**C**), conventional DE analysis (**E**). Each point represents a gene; red indicates significantly differentially expressed genes, gray indicates non-significant genes, and black highlights identified genes of interest. The x-axis shows log2 fold change, and the y-axis shows –log10 (adjusted p-value). Blue dashed lines indicate significant criteria (|log2 fold change|>1.5 and adjusted p-value<0.05). Labelled genes represent our identified candidate ND genes. (**B, D, F**) Scatter plots comparing mean expression levels between wildtype and *MZoep* embryos by Tangram (**B**), SpaGE (**D**), conventional DE analysis (**F**). Genes with higher expression in *MZoep* embryos are shown in red, genes with lower expression are shown in blue, and our identified candidate ND genes are annotated. The diagonal line represents equal expression between conditions. Each plot reports the total number of upregulated and downregulated genes identified by each method. While a comparable fraction (10/13) of the ND genes was recovered using SpaGE-based spatial predictions, Tangram-based predictions and conventional scRNA-seq DE analysis detected only a single ND gene.

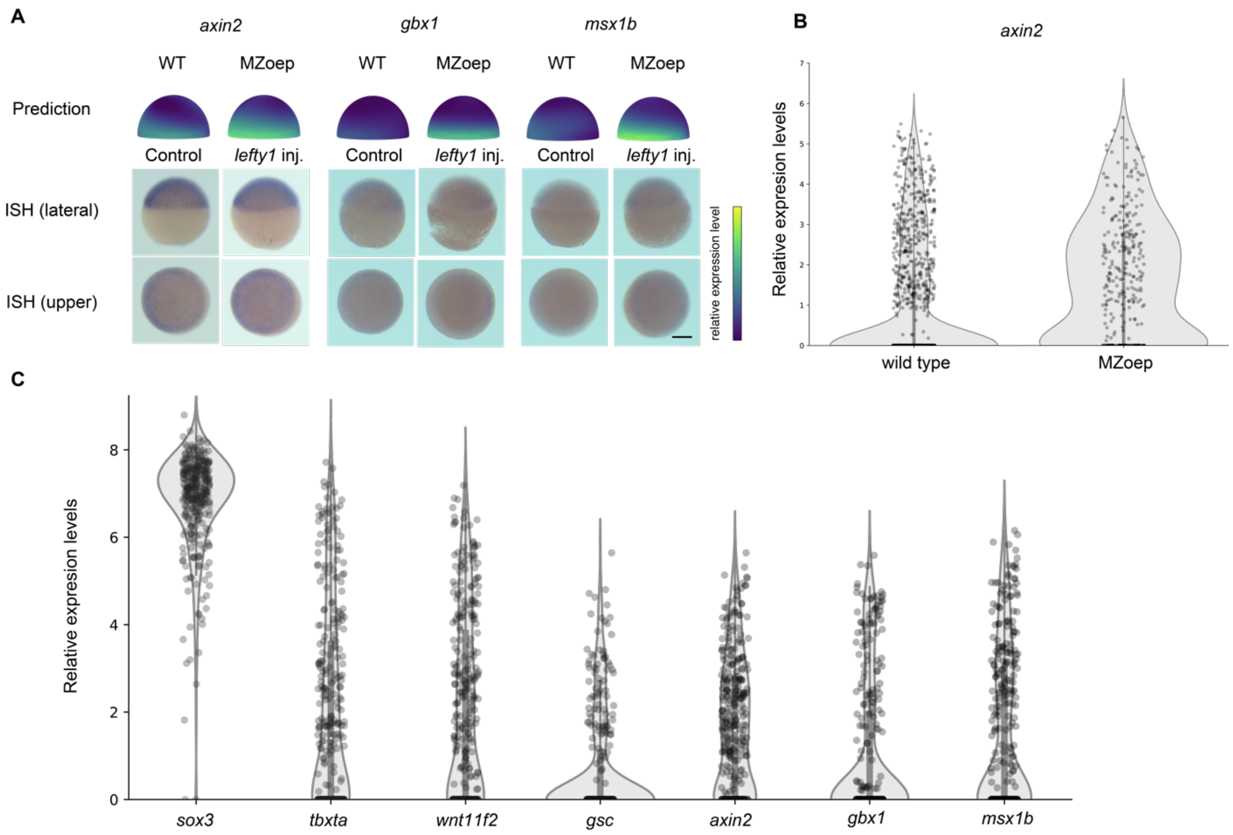

Figure S22. Putative ND genes inconsistent with those of ISH experiments

(A) Whole-mount ISH experiments for *axin2*, *gbx1*, and *msx1b*. From top to bottom: ZENomix-predicted expression pattern, ISH lateral view, and upper view of ISH. For each gene, wild-type and mutant expression patterns are displayed (ZENomix prediction: wild-type and *MZoep*-mutant embryos; ISH experiment: control and *lefty1*-injected embryos). Scale bar, 200 µm. **(B)** Violin plot showing the differences in *axin2* expression between wild type and MZoep scRNA-seq data. scRNA-seq data showed that MZoep embryos had higher *axin2*-expression levels than did wild-type embryos. **(C)** Violin plots of *axin2*, *gbx1*, and *msx1b* expression levels. *sox3, tbxta, wnt11f2,* and *gsc* expression levels are shown in the references. *axin2*, *gbx1*, and *msx1b* are moderately expressed when compared to genes showing low expression (*gsc*) and those showing high expression (*sox3, tbxta*, and *wnt11f2*). Figure 4 shows the ISH images of *gsc*, *sox3, tbxta*, and *wnt11f2*.

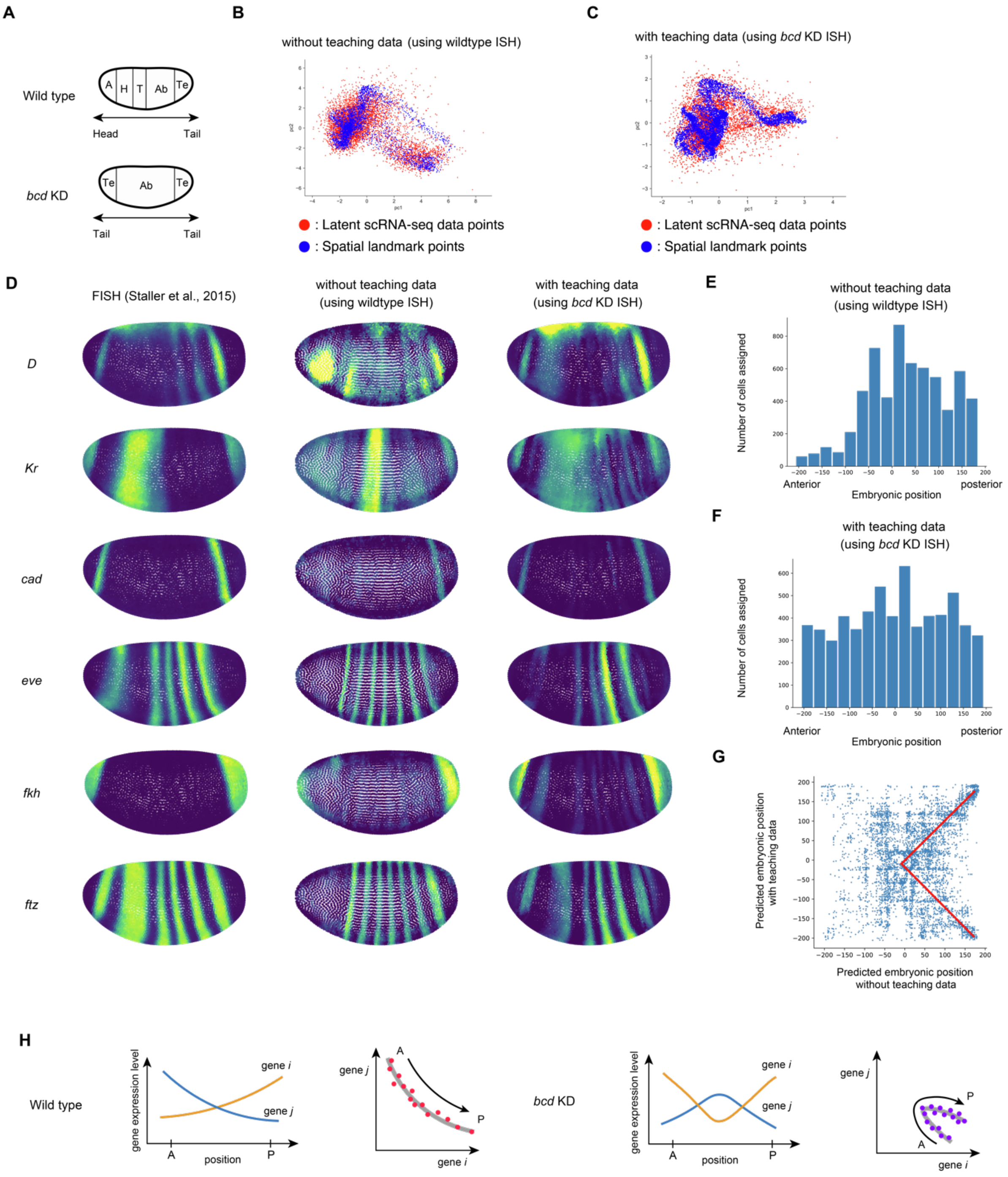

Figure S23. ZENomix application to *bcd*-KD *Drosophila* embryos

(A) Wild-type and *bcd*-KD *Drosophila* embryo phenotypes. The anterior region of the KD embryo is lost and converted to the posterior region. ‘A’, ‘H’, ‘T’, ‘Ab’, and ‘Te’ indicate the acron, head, thorax, abdomen, and telson, respectively. **(B, C)** Scatter plots of matched data point distributions of wild-type spatial reference and *bcd*-KD scRNA-seq data (**B**) and *bcd*-KD spatial reference and *bcd*-KD scRNA-seq data (**C**) (corresponding to **Figure 2b**). Principal component analysis was used to visualise shared latent spaces. (**D**) The *bcd*-KD embryo spatial transcriptome experiment and prediction. We used the FISH data of *bcd*-KD embryos reported by Staller et al. for the experimental data[33]. **(E, F)** Estimated origin of *bcd*-KD scRNA-seq data points when referencing wild type (**E**) and *bcd*-KD *in situ* (**F**) data. The x- and y-axes indicate the estimated embryonic position and number of data points, respectively. (**G**) Relationship between the estimated origin of *bcd*-KD scRNA-seq data points when referencing the *in situ* data of wild-type (**E**) and *bcd*-KD embryos (**F**). The red line structure indicates the symmetric conversion of embryonic structures in the *bcd*-KD embryo, as depicted in **A**. **(H)** The Simple, one-dimensional model of wild type and *bcd*-KD tissues. The blue and orange lines indicate genes i and j expression profiles,

respectively. In the gene expression space, the mutant trajectory corresponds to the posterior part of the wild-type trajectory.

Supplemental Tables

Table S1. Putative NU and ND genes

NU genes							
**Module 1**	*AGRP2*	*ANO9A*	*BX927193.1*	*CABZ01001464.1*	*CABZ01084942.1*	*CR759907.1*	*DIABLOA*
	EIF3J	*ENSDART00000138896*	*FNIP1*	*HNRNPA0B*	*LYRM1*	*MYO3A*	*NME2A*
	RAPGEF6	*SI:CH211-218D20.10*	*SI:CH211-234C11.3*	*SI:CH211-241E1.5*	*SI:CH73-195I19.3*	*SI:DKEY-15N5.6*	*SNAI2*
	TOP1	*UCP3*	*ZGC:171775*	*ZGC:173587*	*ZGC:175284*	*AGRP*	
**Module 2**	*ACVR1BA*	*APLNRA*	*APLNRB*	*BMP2B*	*CHD*	*CST3*	*CTH1*
	CYP27C1	*CYP2AA8*	*DACT2*	*DDIT4*	*DKK1B*	*DRL*	*DUSP4*
	EFNB2A	*EFNB2B*	*ETS2*	*FGF8A*	*FLRT3*	*FOXA*	*FOXA2*
	FOXA3	*FP102169.1*	*FSCN1A*	*FZD7A*	*GADD45BA*	*GATA5*	*GATA6*
	GSC	*ID3*	*IER2*	*IRX7*	*ISM1*	*KIRREL3L*	*LFT1*
	LFT2	*LHX1A*	*MESPAB*	*MIXL1*	*MSGN1*	*NDR1*	*NDR2*
	NOTUM1A	*OSR1*	*OTX1A*	*OTX1B*	*P4HA2*	*PCDH8*	*PITX2*
	PLEKHN1	*RND1L*	*SEBOX*	*SI:CH211-139K8.4*	*SI:CH211-209J10.5*	*SI:DKEY-188C14.4*	*SNAI1A*
	SOX32	*VENT*	*VGLL4L*	*WNT8A*	*XBP1*	*ZIC2A*	*ZSWIM5*
**Module 3**	*ADMP*	*FOXB1A*	*FOXD3*	*HER1*			
**Module 4**	*BMP4*	*BMP7A*	*HES6*	*NOTO*	*TA*	*WNT11F2*	*ZGC:113984*
**ND genes**							
**Module 1**	*CX43*	*MPL*	*SMC1A*				
**Module 2**	*AXIN2*	*CDX4*	*CXCR4A*	*CXCR4B*	*EVE1*	*FOXD5*	*GBX1*
	MSX1B	*SOX19A*	*SOX2*	*SOX3*	*SP5L*	*SZL*	
**Module 3**	*APOC1*	*CLIP4*	*DUT*	*FOXI1*	*HER7*	*OTUD4*	*RAB34B*
	RGCC	*RPL6*	*SI:CH1073-190K2.1*	*SI:CH1073-325M22.2*	*SI:CH211-113A14.16*	*SI:CH211-130H14.6*	*SI:CH211-195B11.4*
	SI:CH211-267E7.11	*SI:CH211-272F15.5*	*SI:DKEY-16M19.4*	*SI:DKEY-23A13.4*	*SI:DKEY-6D5.4*	*SI:DKEY-95P16.2*	*TSR2*
	TWSG1	*SI:DKEY-108K21.12*	*SI:DKEY-261M9.17*	*SI:DKEYP-3B12.8*			

Table S2. Parameter values used in this study

	p	q	The numbers of Inducing points	Initial values of s^2	Initial values of σ^2	Initial values of σ^f_k	σ^f_l	Optimization	RFF
AD-mutant Mouse OB (Figure 2)	62	30	40	1	0.1	0.01	0.01	lbfgs	–
Wild-type Mouse OB (Figure S5)	62	30	40	1	0.1	0.01	0.01	lbfgs	–
MZoep Zebrafish (Figures 3–5)	47	20	40	1	0.1	0.01	0.01	lbfgs	–
Wild-type Zebrafish (Figures 3–5)	47	20	40	1	0.1	0.01	0.01	lbfgs	–
bcd KD Drosophila (without teaching data) (Figure S22)	67	60	100	1	0.1	0.01	0.01	lbfgs	–
bcd KD Drosophila (with teaching data) (Figure S22)	13	7	100	1	0.1	0.01	0.01	lbfgs	–
Wild-type Drosophila (Figure S3)	67	60	100	1	0.1	0.01	0.01	lbfgs	–
PFC_visium (Figure S7–8, S10–13)	1532	50	50	1	0.1	0.01	0.01	adam (0.1, 500)	2048
MTG_merfish (Figure S7–8, S11–12)	140	100	30	1	0.1	0.01	0.01	adam (0.1, 500)	2048
PFC_xenium (Figure S7–8, S11–12)	179	50	50	1	0.1	0.01	0.01	adam (0.1, 500)	2048

For Adam optimizer, learning rate and number of steps are also described.
